# Supplementary material for: Strategies for integrating disparate social information
Source: Proc Biol Sci. 2020 Nov 25;287(1939):20202413. doi: 10.1098/rspb.2020.2413 (PMC7739494; doi:10.1098/rspb.2020.2413)
Supplement: Supplementary Information [file rspb20202413supp1.pdf]

Supplementary Information

Belonging to

Strategies for integrating disparate social information

Lucas Molleman, Alan N. Tump, Andrea Gradassi, Stefan M. Herzog, Bertrand Jayles, Ralf H.J.M. Kurvers and Wouter van den Bos

Proceedings of the Royal Society B: Biological Sciences (2020)

DOI: 10.1098/rspb.2020.2413

Contents

1. Supplementary Figures
2. Supplementary Tables
3. Supplementary Methods
    a) Experimental Methods
    b) Cognitive Model
    c) Simulations
4. Experimental Materials
5. Supplementary References

1. Supplementary Figures

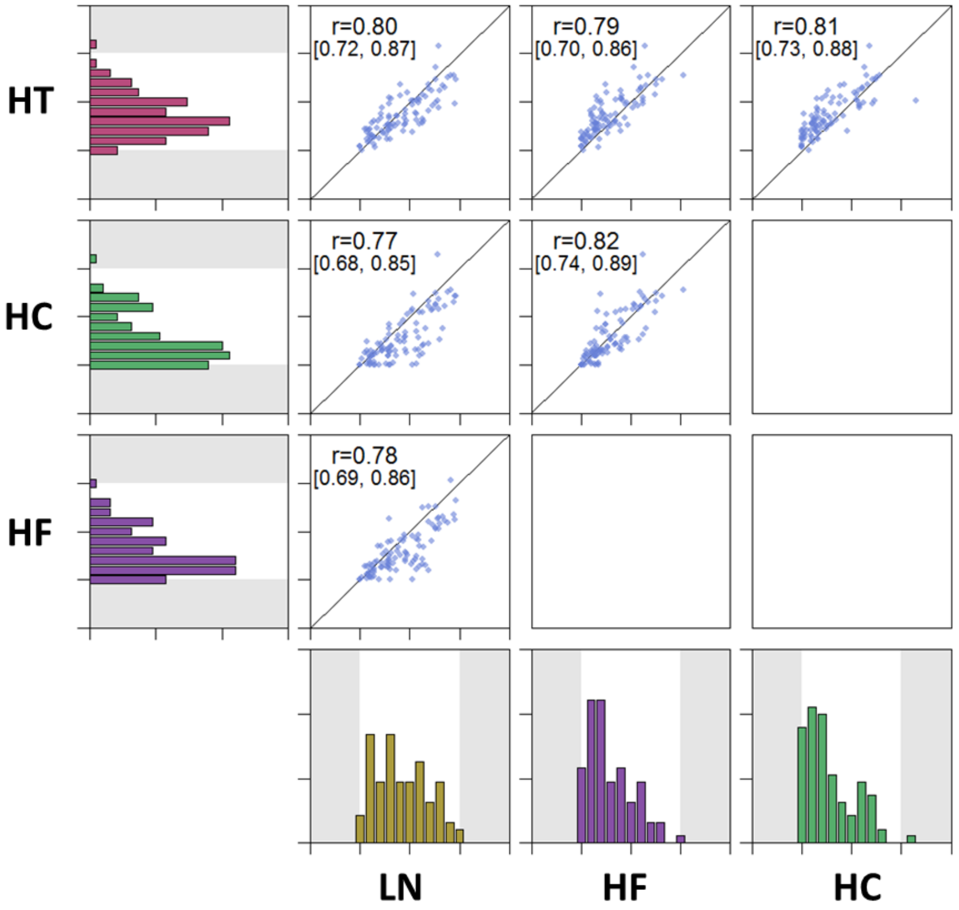

Fig. S1. Distributions of participants' mean adjustment and correlations across the four experimental conditions. Outer histogram panels show frequency distributions of participants' mean adjustments ($\bar{s}$) towards the mean social information for each condition (LN: low variance, no skewness; HN: high variance, no skewness; HF: high variance, cluster of two peers relatively far from the participants first estimate; HC: high variance, cluster of two peers relatively near to the participant's first estimate). Left (right) grey areas in each panel represent values of $\bar{s}$ below 0 (above 1). Across conditions, almost all participants had, on average, an $\bar{s}$ value between 0 and 1, implying they did not adjust away from the social information (i.e., $\bar{s} < 0$), nor adjust beyond the mean social information (i.e., $\bar{s} > 1$). Scatter plots show correlations between participants' mean $\bar{s}$ across the respective conditions. Dots represent participants, and the top left of each panel shows Pearson's *r*, with 95% credible intervals (CI) in square brackets. Overall, we find strong correlations between participants' mean adjustments across conditions, indicating consistent inter-individual differences in social information use. Furthermore, participants' mean adjustments averaged across all conditions correlated in the expected directions with self-reported questionnaire scales measuring conformity (Pearson's *r*=0.26, CI=[0.06, 0.44]), individualism (*r*=-0.20, CI=[-0.39, 0.00]), and resistance to peer influence (*r*=-0.23, CI=[-0.42, -0.03]), in line with previous findings using this paradigm [1].

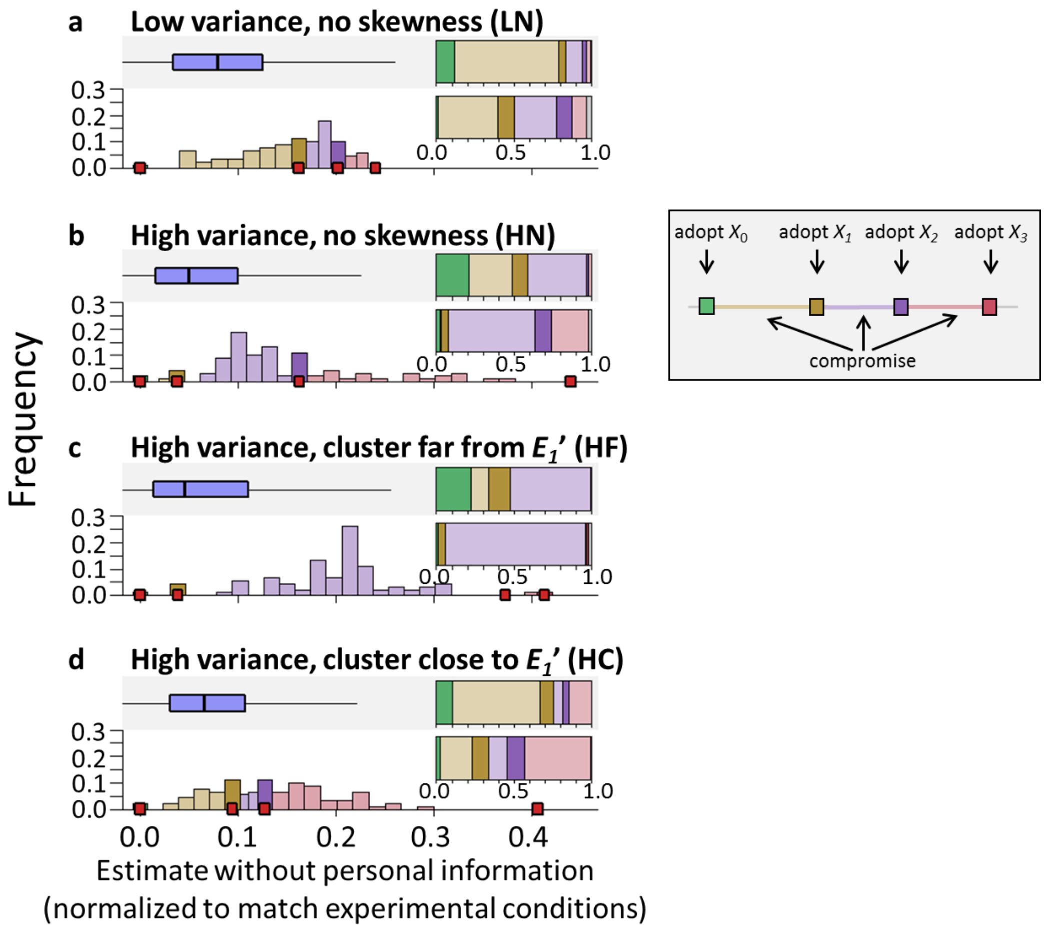

Fig. S2. Adjustments in control conditions in which participants did *not* observe the stimulus, but only observed four peer estimates. Trials were created by first drawing one estimate (E_1') at random from the pool of pre-recorded estimates. Then, three additional pre-recorded estimates were drawn analogous to the four experimental conditions (see Methods). Before plotting, data was normalized in the same way as Fig. 2a-d, taking E_1' as the reference point. Histograms show distributions of estimates in the same colour coding as Fig. 2a-d. For comparison, in the grey background on top of each panel we summarize behaviour in conditions where participants *did* observe the stimulus (cf. Fig. 2a-d; blue boxplot: distributions of relative estimates; insets: relative frequencies of each of the qualitative cases).

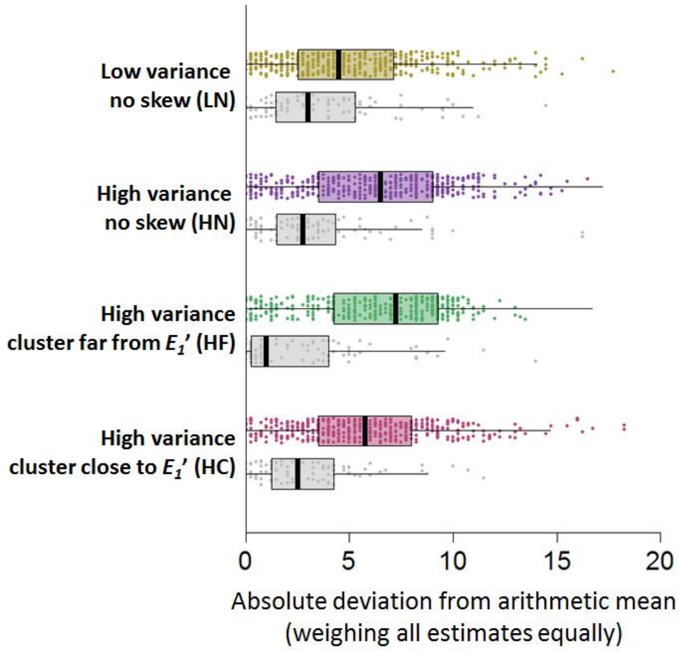

Fig. S3. Both in experimental (i.e., with observing the stimulus; coloured dots and boxplots) and control (i.e., without stimulus; grey dots and boxplots) conditions, participants systematically deviated from an 'ideal Bayesian observer', who would weigh all estimates equally. Boxplots and dots show the absolute difference between participants' second estimates and the arithmetic mean of all four estimates (see Methods more details). Participants deviated more from the arithmetic mean in the experimental conditions compared to the control conditions.

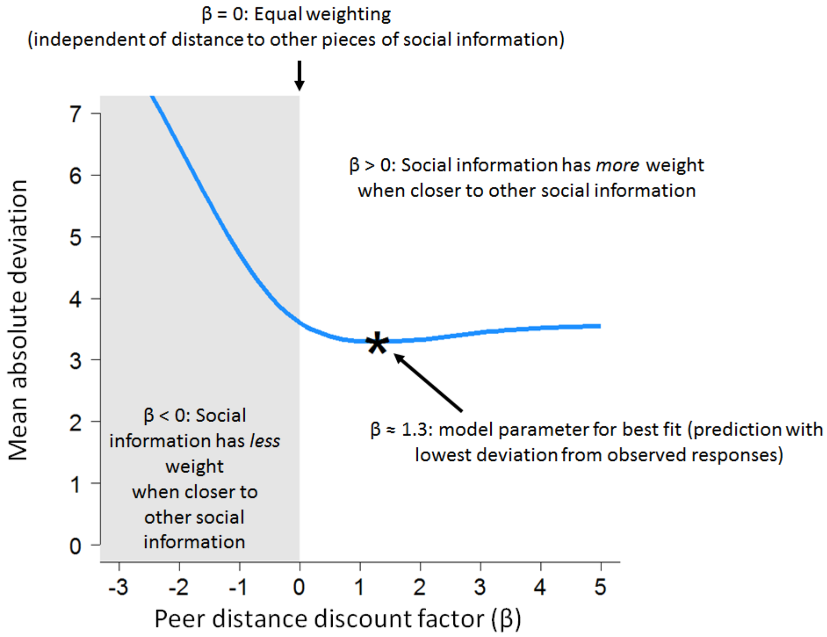

Fig. S4. Proximity-based weighting can account for responses (E_2') in the control rounds in which participants did not observe the stimulus. The blue line shows the mean absolute difference between per-round predictions and observed responses as a function of parameter β (capturing proximity-based weighting as the extent of discounting of social information that is inconsistent with other social information). This model assumes that each response is an average of the pieces of social information (X_i), weighted according to their summed distance (d) to other pieces of social information. Formally, predicted responses are calculated as: $\widehat{E_2}' = \sum_{i=1}^{4} w_i \cdot X_i$. In this formula, the weighting of each piece of social information (w_i) is determined by discount factor β: $w_i = \frac{{d_i}^{-\beta}}{\sum_{j=1}^{4} {d_j}^{-\beta}}$, where $d_i = \sum_{j=1}^{4} |X_i - X_j|$. When β > 0, more weight is assigned to social information when it is closer to other social information. We observe that the model predictions match the observed responses best when β ≈ 1.3. We interpret this as evidence of proximity-based weighting: on average, participants tend to assign more weight to social information when it is closer to other social information.

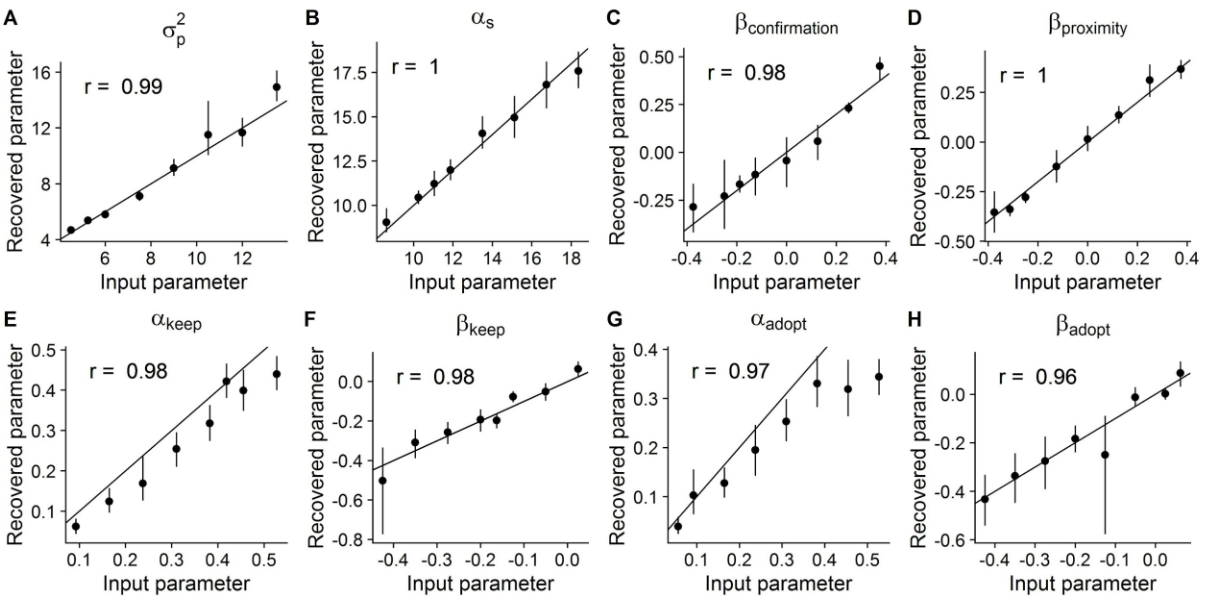

Fig. S5. Recovery of all parameters of the full model. The dots and error bars indicate mean and the 95% CI of the posterior mean estimates. The diagonal line indicates the position expected under perfect parameter recovery. For the majority of recovered parameter estimates, the generating (input) parameters lie within the 95% CI. This indicates that the model parameters are identifiable. The parameter estimates for α'_{keep} and α'_{adopt} are lower than the generating parameter values. This is a result of choosing priors for the group-level parameters reflecting our expectation that high probabilities to keep or adopt are less likely (see Table S4). In each panel, numbers in the top left corner indicate Spearman's correlation coefficient.

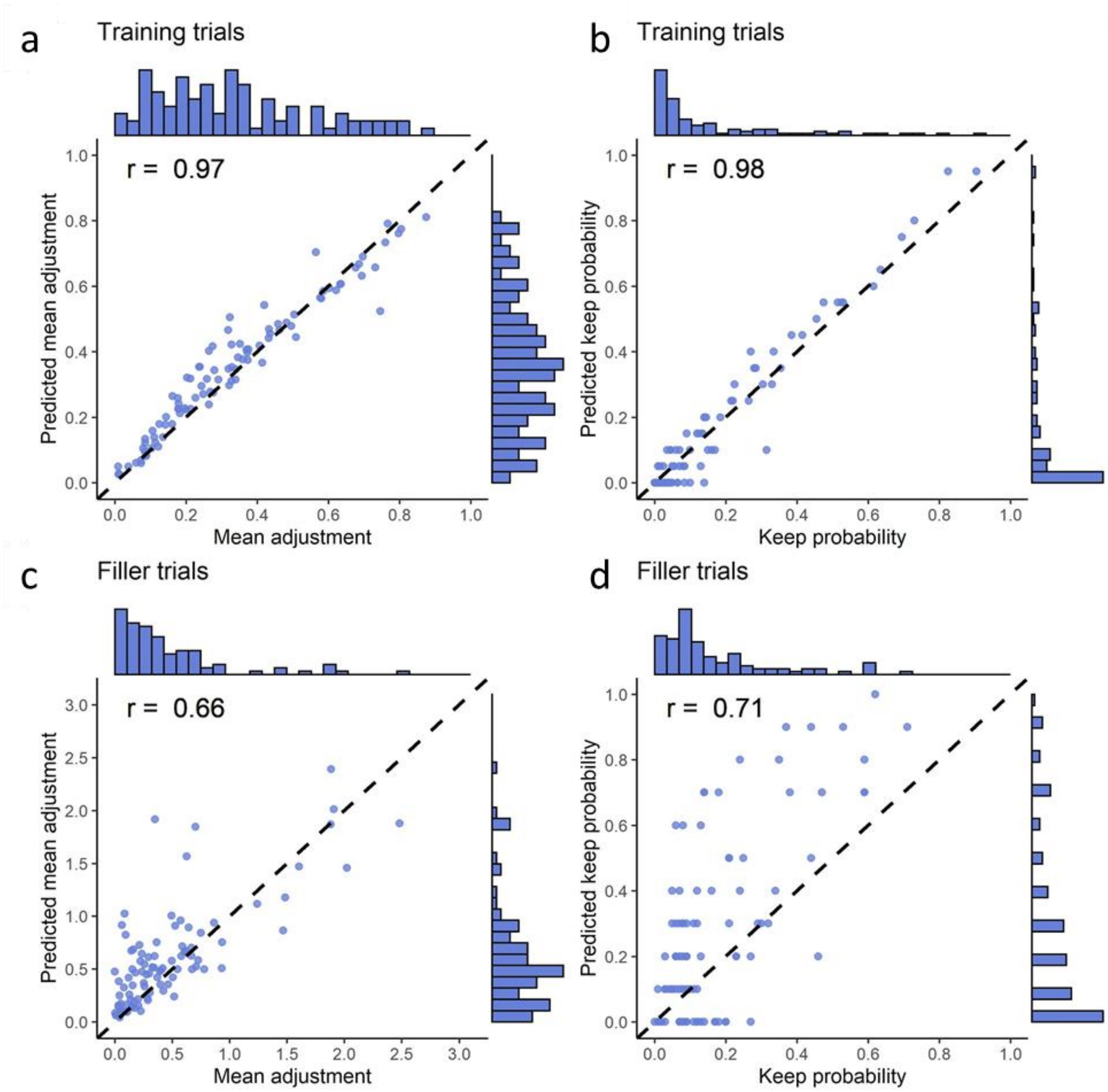

Fig. S6. Correlation of predictions of the best-fitting cognitive model (y-axis) with the empirical data (x-axis). **a-b,** Predictions for the 20 rounds of the 4 experimental conditions, to which the model was fitted (i.e. the 'training set'). **a,** Mean adjustment for each participant. **b,** Fraction of rounds in which they chose to choose to 'keep' their first estimate. We observe that the predictions of the cognitive model closely match data in the experimental conditions. **c-d,** Predictions for the 'filler' trials with social information randomly drawn from previous participants (on which the model was not trained). Also for these out-of-sample predictions, the model quite closely tracks the empirical data. As for previous predictions we sampled 10 estimates for each participant and trial. In each panel, numbers in the top left corner indicate Pearson's correlations.

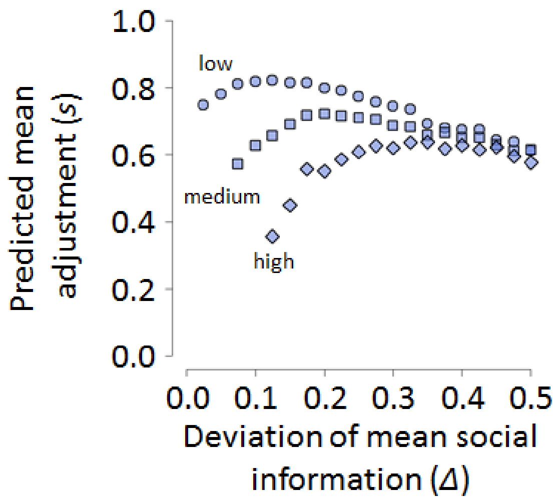

Fig. S7. Social information tends to have the strongest impact when at intermediate distance. We simulated trials that systematically varied the distance between individuals' own first estimates and the mean social information ($\Delta = |\bar{X} - E_1| \, / \, E_1$). Dots show predicted mean adjustments as a function of the mean distance to three pieces of social information, for three levels of 'peer proximity', with peer estimates at 0, 3 or 6 numbers away from each other (respectively reflecting 'low', 'medium' or 'high' variance). We observe that mean shifts for each level are highest when the distance of social information is intermediate. This shows that our model is able to recover key results from previous studies showing this nonlinear effect of the distance of social information [2,3]. When the distance to mean social information is very low, individuals are very likely to keep their first estimates. When the distance to mean social information is very high, individuals are likely to compromise, but assign little weight to social information, again leading to reduced average adjustments. At intermediate distance, social information has the strongest impact: in those cases, the likelihood that individuals keep their first estimates is very close to zero, but when compromising, they still assign positive weight to social information. These effects hold across various levels of peer proximity. As expected, lower variance in social information leads to larger mean adjustments. Note that simulations with peers farther away from each other (i.e. 'high') start at higher values of Δ to avoid that the social information brackets the own first estimate. Including these 'bracketed' cases would show nonlinear adjustment patterns, because near Δ=0 the distance to the nearest peer is a nonlinear function of Δ.

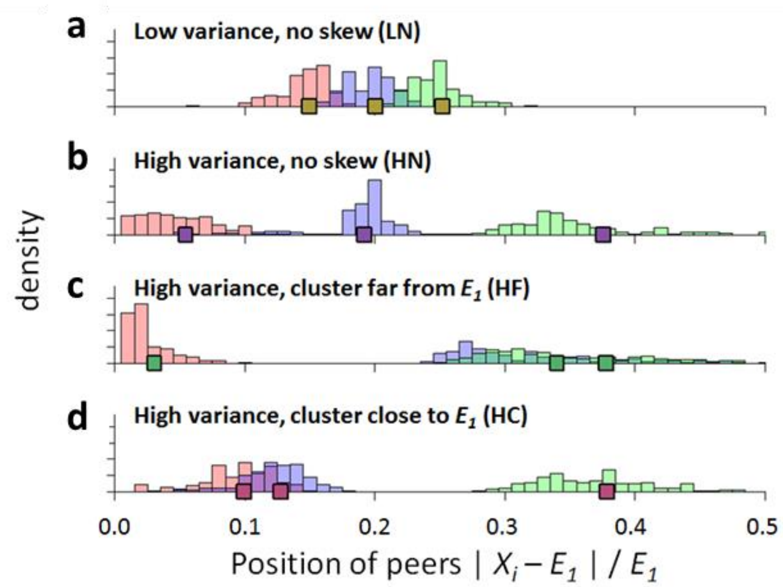

Fig. S8. Relative position of peers as shown in each of the experimental conditions. Social information was based on data from a pre-recorded pool of 100 MTurkers who completed the task without social information. We assigned an experimental condition to each of the 30 rounds and calculated for each value of E_1 the 'triple' of previous estimates that most closely matched the experimental condition (see below, Section 3a, 'Definition of experimental conditions', for a formal description). The graph shows for each condition where the peers were located in the actual experiment, expressed as their absolute deviation from E_1, divided by E_1. Colour coding: red: nearest peer, blue: middle peer, green: farthest peer. Coloured squares show for each of the three peers their average position (cf. Fig. 1e).

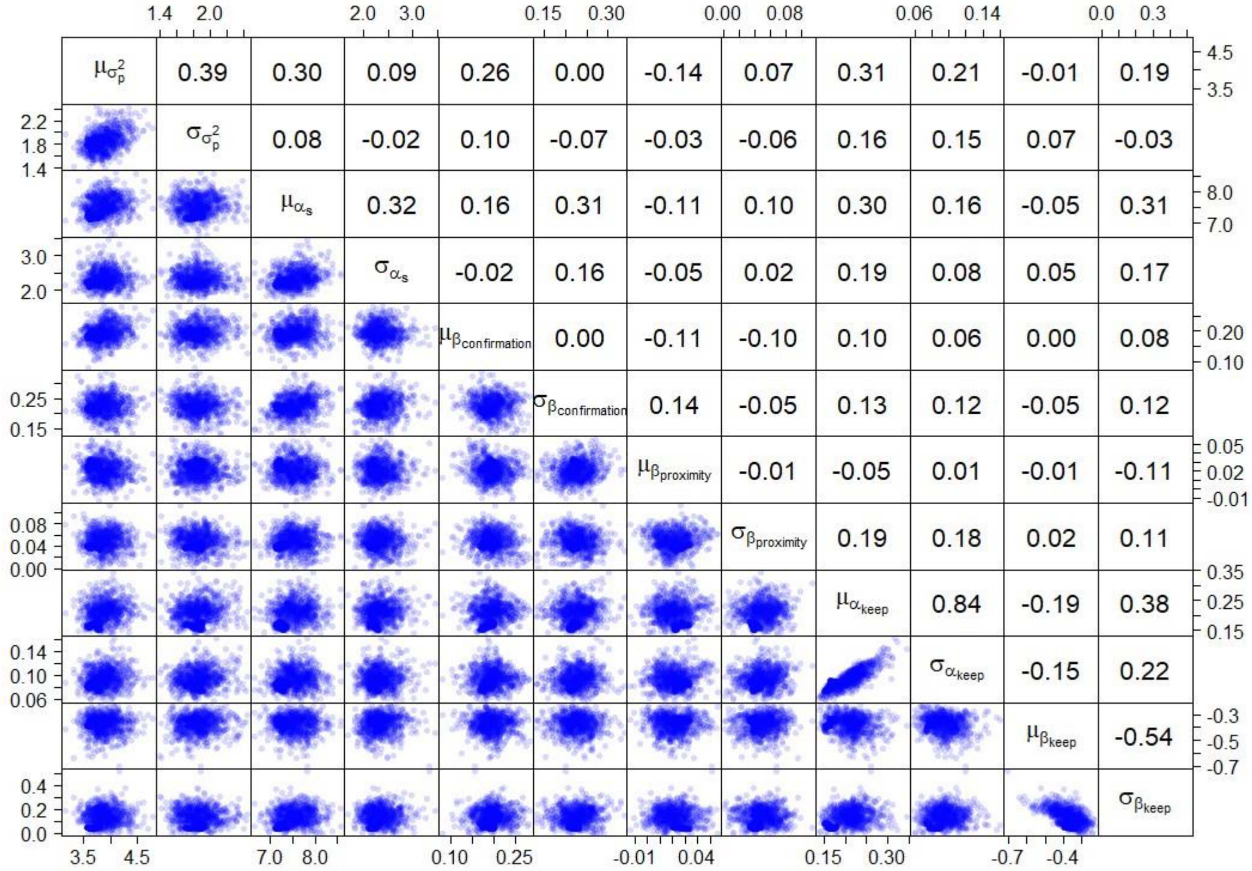

Fig. S9. The lower left side shows the joint posterior samples of the group-level means of the best fitting model. The upper right side displays Pearson correlation coefficient among these parameters. We find correlations between some mean and corresponding variance parameters. Apart from this finding, we do not find very strong correlations among the parameters indicating that the parameters are relatively independent (suggesting that the marginal distributions of the parameters can be interpreted in isolation). To have comparable measures for all parameters we display the mean and variance of the group-level beta distribution of α'_{keep} via: $\mu = \frac{\alpha}{\alpha+\beta}$, $\tau = \frac{\alpha\beta}{(\alpha+\beta)^2\,(\alpha+\beta+1)}$.

2. Supplementary Tables

Table S1. Determinants of participants' social information use. Numbers on the left hand side show results from a Bayesian linear mixed model (LMM; fitted with the R-package *brms* [4] version 2.13.5, using default priors) fitted to participants' average adjustments towards the mean social information across the rounds of each of the experimental conditions, with 'participant' as varying intercept. Numbers between brackets indicate 95% posterior credible intervals. On the right-hand side, we show pairwise comparisons between experimental conditions. Average adjustments were calculated as the relative distance adjusted towards the mean social information (Fig. 1d), across the five rounds of each of the four experimental conditions (LN, HN, HF, HC; Fig. 1e), yielding four data points for each participant. We omitted those rounds in which a participant adjusted away from the mean social information. The LN condition was used as the baseline. Relative to that baseline, the HN and HF conditions had a strong negative effect. The HC condition had a smaller negative effect. Pairwise comparisons indicate that, except for the HN and HF conditions, all pairs of experimental conditions differed significantly from each other. Age and gender did not seem to affect social information use (the 95% CI shown for 'age' is rounded from [-0.004, 0.004]). To quantify the participants' constancy of displayed behaviour across conditions we also derived the commonly used index of 'repeatability' for this model (R=0.79 [0.70, 0.84]) [5]. Finally, an LMM fitted to adjustments in individual rounds, with 'round' as an additional predictor, confirms the results shown here, and reveals that adjustments did not change over the course of the experiment (see Table S7).

	**Average adjustment**		
	**Estimate [95% CI]**	**Pairwise comparison**	**Estimate [95% CI]**
Low variance, No skew (LN); baseline	0.39 [0.20, 0.57]	HN - LN	-0.13 [-0.15, -0.10]
High variance, No skew (HN)	-0.13 [-0.16, -0.09]	HF - LN	-0.14 [-0.16, -0.11]
High variance, cluster far from *E1* (HF)	-0.14 [-0.17, -0.10]	HC - LN	-0.05 [-0.08, -0.02]
High variance, cluster close to *E1* (HC)	-0.05 [-0.08, -0.02]	HF - HN	-0.01 [-0.03, 0.02]
Age	0.00 [-0.00, 0.00]	HC - HN	0.08 [0.05, 0.10]
Gender (0=male, 1=female)	0.02 [-0.07, 0.12]	HC - HF	0.09 [0.06, 0.11]
N	95		
n	380		

Table S2. Marginal effects of experimental conditions on participants' use of adjustment strategies. Values indicate for each condition the predicted relative frequencies of adjustment strategies - keeping, compromising, adopt the nearest peer (X_1), and all 'other' cases pooled - from a mixed multinomial regression with 'participant' as varying intercept (cf. insets Fig. 2a-d of the main text), fitted with the R-package *brms*[4] version 2.13.5, using default priors. Values in brackets indicate 95% posterior credible intervals. Apart from the experimental conditions, the regression model also included age and gender, neither of which had credible non-zero effects on any of the relative frequencies.

condition	keep	compromise	copy X1	other
**LN**	0.07 [0.04, 0.10]	0.79 [0.70, 0.86]	0.02 [0.01, 0.05]	0.12 [0.07, 0.19]
**HN**	0.16 [0.11, 0.22]	0.26 [0.18, 0.37]	0.07 [0.04, 0.11]	0.51 [0.37, 0.63]
**HF**	0.16 [0.11, 0.23]	0.08 [0.05, 0.13]	0.09 [0.05, 0.15]	0.67 [0.54, 0.77]
**HC**	0.06 [0.04, 0.09]	0.67 [0.56, 0.76]	0.06 [0.03, 0.10]	0.21 [0.13, 0.32]

Table S3. The comparison of all versions of the cognitive models with all possible combinations of considered features. We compared models with (1) and without (0) each of the four features (keeping, adopting, confirmation-based and proximity-based weighting) and calculated the looic. The differences in expected log pointwise predictive density ('elpd_diff') indicate the expected predictive accuracy of each model compared to the model with the lowest looic (i.e., rank 1), with 'se_diff' indicating the corresponding standard error. The best-fitting model includes the keep heuristic, confirmation- and proximity-based weighting, but not the 'adopt' heuristic. Note that the model including 'adopt' (i.e., rank 2) has only marginally lower predictive accuracy. Inspection of the results from the full model (including all features) reveals that predicted probabilities of adopting are very low.

Keep	Adopt	Proximity	Confirmation	Looic	elpd_diff	se_diff	Rank
1	0	1	1	8222	0	0	1
1	1	1	1	8225	-1.55	3.62	2
1	0	0	1	8237	-7.66	5.52	3
1	1	0	1	8247	-12.61	6.37	4
1	1	1	0	8433	-105.62	16.71	5
1	0	1	0	8444	-111.27	16.25	6
1	1	0	0	8479	-128.57	17.12	7
1	0	0	0	8498	-137.92	16.78	8
0	0	1	1	8713	-245.72	26.67	9
0	0	0	1	8719	-248.75	26.62	10
0	1	1	1	8720	-249.18	26.97	11
0	1	0	1	8726	-251.91	26.75	12
0	1	1	0	8982	-379.81	30.67	13
0	0	1	0	8995	-386.8	31.12	14
0	1	0	0	9014	-396.15	31.35	15
0	0	0	0	9037	-407.4	31.02	16

Table S4. The parameters shaping the group-level distributions, their priors, their upper (lower) bounds and the model estimates of the best fitting model (i.e, without the 'adopt' heuristic). The parameters μ and τdescribe the mean and standard deviation of group-level normal distributions and α and β the two parameters shaping the group-level beta distributions. Standard deviations and parameters describing the beta distributions were truncated at 0.01 to avoid zero or negative values. Subscripts *p* and *s* respectively indicate personal and social information; the other subscripts refer to their respective model feature (confirmation- or proximity-based weighting; heuristics of keeping or adopting). The right-hand side column shows the parameter estimates of the best-fitting model, with brackets indicating 95% posterior credible intervals. This model did not include the 'adopt' heuristic (see Table S3) so no values are shown for the parameters associated with that heuristic.

Group-level parameters	Priors	Bounds (max/min)	Parameter estimates
$\mu_{\sigma_p^2}$	N(10, 5)	1.0 / Inf	3.91 [3.50, 4.40]
$\tau_{\sigma_p^2}$	N(0, 1)	0.01 / Inf	1.87 [1.54, 2.30]
μ_{α_s}	N(10, 5)	1.0 / Inf	7.53 [6.91, 8.20]
τ_{α_s}	N(0, 1)	0.01 / Inf	2.35 [1.92, 2.87]
$\mu_{\beta_{confirmation}}$	N(0, 0.2)	-0.5 / 0.5	0.20 [0.13, 0.26]
$\tau_{\beta_{confirmation}}$	N(0, 0.2)	0.01 / Inf	0.22 [0.15, 0.29]
$\mu_{\beta_{proximity}}$	N(0, 0.2)	-0.5 / 0.5	0.02 [0.00, 0.04]
$\tau_{\beta_{proximity}}$	N(0, 0.2)	0.01 / Inf	0.05 [0.01, 0.08]
$\alpha_{\alpha'_{keep}}$	N(1, 0.4)	0.01 / Inf	0.18 [0.12, 0.30]
$\beta_{\alpha'_{keep}}$	N(5, 2)	0.01 / Inf	0.71 [0.45, 1.11]
$\mu_{\beta_{keep}}$	N(0, 0.5)	-Inf / Inf	-0.36 [-0.52, -0.25]
$\tau_{\beta_{keep}}$	N(0, 1)	0.01 / Inf	0.14 [0.02, 0.30]
$\alpha^{\alpha'_{adopt}}$	N(1, 0.4)	0.01 / Inf	
$\beta_{\alpha'_{adopt}}$	N(5, 2)	0.01 / Inf	
$\mu_{\beta_{adopt}}$	N(0, 0.5)	-Inf / Inf	
$\tau_{\beta_{adopt}}$	N(0, 1)	0.01 / Inf	

Table S5. Pearson correlations for the mean posterior parameter estimates across participants of the best-fitting model, with 95% posterior credible intervals in brackets. Overall, we did not find strong correlations between pairs of parameter estimates, with three exceptions: (*i*) A low weight on own first estimates (higher ${\sigma^2}_p$) is associated with stronger proximity-based weighting (higher $\beta_{proximity}$) and (*ii*) a lower tendency to keep their first estimates (lower α'_{keep}). (*iii*) A stronger tendency to keep first estimates (higherα'_{keep}) is associated with a lower sensitivity to the distance of the nearest peer (less negative β_{keep}).

	σ_p^2	α_s	$\beta_{confirmation}$	$\beta_{proximity}$	α_{keep}	β_{keep}
σ_p^2	1.00					
α_s	-0.02 [-0.22, 0.18]	1.00				
$\beta_{confirmation}$	-0.13 [-0.32, 0.07]	0.00 [-0.20, 0.20]	1.00			
$\beta_{proximity}$	**0.37 [0.18, 0.53]**	-0.13 [-0.32, 0.08]	-0.10 [-0.30, 0.10]	1.00		
α_{keep}	**-0.21 [-0.40, -0.01]**	0.02 [-0.19, 0.22]	0.12 [-0.09, 0.31]	0.00 [-0.20, 0.20]	1.00	
β_{keep}	-0.01 [-0.21, 0.19]	-0.01 [-0.21, 0.19]	0.02 [-0.19, 0.22]	0.00 [-0.20, 0.20]	**0.37 [0.18, 0.53]**	1.00

Table S6. Description of the model parameters and the parameters of their group-level distribution.

**Model feature**	**Parameter**	**Group-level distributions**	**Description**
**Keep**			
Keep intercept	α'_{keep}	$Beta(\alpha_{\alpha'_{keep}}, \beta_{\alpha'_{keep}})$	A value between zero and one describing the baseline probability of keeping the first estimate.
Keep slope	β_{keep}	$N(\mu_{\beta_{keep}}, \tau_{\beta_{keep}})$	The influence of the distance of the nearest peer on the probability to keep the first estimate.
**Adopt**			
Adopt intercept	α'_{adopt}	$Beta(\alpha_{\alpha'_{adopt}}, \beta_{\alpha'_{adopt}})$	A value between zero and one describing the baseline probability of adopting the estimate of a peer.
Adopt slope	β_{adopt}	$N(\mu_{\beta_{adopt}}, \tau_{\beta_{adopt}})$	The influence of distance on the probability to adopt the estimate of a peer.
**Compromise**			
Uncertainty first estimate	${\sigma^2}_p$	$N(\mu_{{\sigma^2}_p}, \tau_{{\sigma^2}_p})$	Uncertainty associated with personal information (i.e. the first estimate, E_1)
Intercept uncertainty peer estimate	α_s	$N(\mu_{\alpha_s}, \tau_{\alpha_s})$	The intercept uncertainty associated with the peer estimates (X_i)
Confirmation-based weighting	$\beta_{confirmation}$	$N(\mu_{\beta_{confirmation}}, \tau_{\beta_{confirmation}})$	The influence of closeness of the peer estimate to the first estimate on the uncertainty associated with the peer estimate
Proximity-based weighting	$\beta_{proximity}$	$N(\mu_{\beta_{proximity}}, \tau_{\beta_{proximity}})$	The influence of proximity of the peer estimate to other peers on the uncertainty associated with the peer estimate

Table S7. Determinants of participants' social information use in individual rounds of the experiment. Estimates show results from a Bayesian linear mixed model (LMM; fitted with the R-package *brms* [4] version 2.13.5, using default priors) fitted to participants' adjustments towards the mean social information in the rounds of each of the experimental conditions, with round number, experimental condition (LN, HN, HF or HC), age and gender as predictors. We further included 'participant' as varying intercept, and random slopes for round number. Numbers between brackets indicate 95% posterior credible intervals (the 95% CI shown for 'round' is rounded from [-0.001, 0.002], and for 'age' from [-0.004, 0.004]). We observe that 'round' does not predict social information use, suggesting that over time, individuals did not change their social information use. Treatment effects are very similar to those reported in Table S1.

	Estimate	95% CI
**round**	0.00	[-0.00, 0.00]
**Low variance, No skew (LN); baseline**	0.36	[0.18, 0.55]
**High variance, No skew (HN)**	-0.13	[-0.16, -0.10]
**High variance, cluster far from E1 (HF)**	-0.14	[-0.16, -0.11]
**High variance, cluster close to E1 (HC)**	-0.05	[-0.08, -0.02]
**Age**	0.00	[-0.00, 0.00]
**Gender (0=male, 1=female)**	0.04	[-0.06, 0.13]
**N**	95	
**n**	1839	

3. Supplementary Methods

3a. Behavioural experiment

General procedures and experimental paradigm. We recruited 100 participants from Amazon Mechanical Turk (MTurk), restricted to US citizens, and to MTurkers with a minimum approval rate of 95%. By clicking the link to the experimental pages, participants confirmed informed consent. Five participants dropped out during the task and did not receive any payment, resulting in 95 participants (57% male, mean ± s.d. age = 36 ± 11 years). The online experiment was programmed in LIONESS Lab [6].

The experimental task is based on a validated perceptual judgment paradigm for quantifying social information use (BEAST [1]; Fig. 1a-c). The basic version of this task has been used in samples from various ages and cultural backgrounds [1,7,8], and has been shown to have high test-retest reliability with participants recruited from MTurk [1]. In each of 30 rounds (5 per experimental condition, plus 10 'filler' rounds with randomly selected social information, see section 'Definition of experimental conditions' below), participants observed an image with 50-100 animals for 6 seconds and had to estimate how many animals there were. They entered their estimates with a slider limited from 1 to 150 (for screenshots, see section Experimental Materials below). After submitting their first estimate (E_1), they observed the estimates of three other participants (X_1, X_2, X_3) who had completed the same task before but without receiving social information. After observing the social information, participants provided a second estimate (E_2). No feedback about performance was given during the task, curbing participants' learning about the accuracy of their own first estimates, or whether their responses to social information led to an improved performance.

Participants were rewarded for accuracy, earning 100 points if their estimate was exactly correct (both for E_1 and E_2). For each animal they were off, five points were subtracted (but earnings in a round could not drop below zero). At the end of the session, one decision was randomly chosen from each of the experimental 'blocks' (see below) for bonus payment (100 points = $1.00), which came on top of a flat fee of $4.50. Total earnings ranged from $4.50 to $7.00 (average $5.50). Participants took, on average, 35 minutes, resulting in an hourly wage of $9.50. Experimental sessions ended with a short questionnaire in which we recorded participants' age and gender, and measured individualism [9], social conformity [10], and resistance to peer influence [11]; see caption of Fig. S1.

To study the effects of disparate social information on behaviour, we used four experimental conditions (Fig. 1e of the main text). Across conditions, we systematically manipulated the variance and skewness of the distributions of social information, while keeping the distance between the mean social information ($\overline{X}$) and E_1 constant. To achieve experimental control without deception, we first recorded a large (N=100) pool of estimates made by MTurkers for each image shown in the main experiment. Like in the main experiment, the MTurkers in this 'pre-test' were rewarded for accuracy: after making their estimates, we randomly selected one estimate for payment (earning 100 points if their estimate was correct; five points were subtracted for each animal off; earnings could not be lower than zero; again, 100 points = $1.00). In a given round of the main experiment, the three pieces of social information were selected based on the participant's first estimate and the experimental condition assigned to that round (for implementation details, see section 'Definition of experimental conditions' below). This procedure resulted in clearly defined experimental conditions (Fig. S7). We randomly shuffled the order of experimental conditions across rounds and held this order fixed for all participants.

Definition of experimental conditions. Participants faced four experimental conditions in which they could adjust their first estimates based on three pieces of social information. These conditions varied in the variance and skewness of the social information (Fig. 1e, main text). For each round, for each possible first estimate (E_1) we considered each possible triple of unique pre-recorded estimates, and calculated the first three moments of its distribution (mean μ, variance σ^2 and skewness γ). To determine which triple would be shown in a given condition in a given round for a given value of E_1, we used a cost function that assigned penalties (L) to deviations from the target mean (T_μ), target variance ($T_\sigma{}^2$) and target skewness (γ). For each round, for each possible value of E_1 we selected the triple with the lowest L. The cost functions L for each condition are given in the below table, which shows the penalties for deviations from the target mean, variance and skewness in separate columns:

**Mean**		**Variance**		**Skewness**
Low variance, no skew (LN)				
$100 \cdot \mid \mu - T_\mu \mid$	+	$10 \cdot \mid \sigma^2 - T_\sigma{}^2 \mid$	+	$10 \cdot \mid \gamma \mid$
High variance, no skew (HN)				
$100 \cdot \mid \mu - T_\mu \mid$	+	$10 \cdot \mid \sigma^2 - T_\sigma{}^2 \mid$	+	$500 \cdot \mid \gamma \mid$

High variance, cluster far from E_1 (HF)				
$100 \cdot \| \mu - T_\mu \|$	+	$10 \cdot \| \sigma^2 - T_\sigma^2 \|$	-	$1{,}000 \cdot \gamma'$
High variance, cluster close to E_1 (HC)				
$100 \cdot \| \mu - T_\mu \|$	+	$10 \cdot \| \sigma^2 - T_\sigma^2 \|$	+	$1{,}000 \cdot \gamma'$

In all conditions, T_μ was set to deviate 20% from E_1. We held this distance fixed to avoid possible effects of the mean deviation of social information on its impact on behaviour [2,3] (see also Fig. S7 for an illustration of how this deviation may affect social information use). For further standardization, T_μ was always in the direction of the true value A[1,12]. Formally, $T_\mu = 1.2 \cdot E_1$ if (E_1>A) and $T_\mu = 0.8 \cdot E_1$ if (E_1<A). We set T_σ^2 =10 for the LN condition and T_σ^2 =100 for all other experimental conditions. For the LN and HN we aimed for symmetric distributions so we penalized positive absolute values of γ. For HF and HC, target skewness depended on whether E_1 was higher or lower than A. To this end, we used γ' which was equal to γ if E_1>A and equal to -γ if E_1<A. This procedure ensured that participants faced well-defined experimental conditions (Fig. S8). For the 10 'filler' rounds, which were intermixed with the experimental rounds, we randomly drew social information from the pre-recorded pool.

We further implemented two control conditions completed in separate blocks of the experimental session (these blocks were completed in randomized order). First, participants completed trials in which they did not observe the stimulus themselves, but only observed the estimates of four peers (Fig. S2). The distribution of these peer estimates emulated the distributions of social information in each of the experimental conditions, enabling us to compare how individuals integrate personal and social information with a control in which individuals integrate four pieces of information, none of which is their first estimate. Second, participants could observe the estimate of only one peer whose deviation from the individuals' first estimate matched that of the mean deviation in the four experimental conditions. The results from this one-peer control condition are not the focus of this paper and will not be reported here.

Participants' belief that social information was real. Estimates in our experiment were incentivised so that participants should only adjust them based on social information in case they think it will improve their accuracy. The fact that overall, adjustments were quite substantial, suggests that participants trusted the social information. Furthermore, in a separate study using a basic version of the BEAST paradigm (manuscript in preparation), we asked participants to elaborate on how they

integrated the estimates of other MTurkers into their own judgments. Out of 209 participants, only three expressed doubt about the veracity of the social information shown to them (and hence chose to ignore it). This suggests that the vast majority of MTurkers tend to trust that the real social information in our experiment was indeed real.

3b. Cognitive Model

In this section we describe and analyse our cognitive model explaining social information use in our experiments. We first formalize our model assumptions below, and then present the model analysis. We conclude this section with robustness checks.

Overview. To gain a detailed understanding of how individuals in our experiment integrated social information, we developed a set of models that combine simple heuristics (keeping and adopting) and more complex strategies (compromising; cf. Fig. 3a). In our model, individuals first select their adjustment strategy ('keep' their own personal estimate, 'adopt' the estimate of the nearest peer, or 'compromise' towards the social information). We assume that an individual's strategy selection depends on the distance between their own first estimate and the estimate of the nearest peer [13,14]. If individuals select the compromising strategy, the weight of each of the peer estimates depends on its distance to an individual's own first estimate ('confirmation-based weighting' [2,3,12,15]), and its distance to other peer estimates ('proximity-based weighting'; Fig. S4 [13,14]).

Our assumptions are reflected in four model features which define the selection of (*i*) the keep heuristic, or (*ii*) the adopt heuristic, and, when compromising, the weighting of social information based on (*iii*) confirmation or (*iv*) proximity. We use Bayesian techniques to examine the effects of each of these model features separately, as well as any combination of features. By identifying the combination of model features that best explain the experimental data, and examining their best-fitting parameters, we obtain a detailed picture of how individuals select an adjustment strategy, and how they implement the weighting of personal and social information.

Choosing an adjustment strategy (*keep*, *adopt* or *compromise*). Individuals choose one of the three strategies with respective probabilities *P*(keep), *P*(adopt), and *P*(compromise). The sum of the three probabilities adds up to 1 [16]. The probability to *keep* is given by the logistic function $P(keep) = [1 + \exp(-K)]^{-1}$, where $K = \alpha_{keep} + \beta_{keep} \cdot d_1$. In this formula, the parameters

α_{keep} and β_{keep} determine the intercept and slope of the logistic function, and d_1 is the absolute distance between the first estimate and the nearest peer estimate: $d_1 = |E_1 - X_1|$. Likewise, we define the probability to *adopt* the estimate of the nearest peer as $P(adopt) = [1 + \exp(-A)]^{-1}$, where $A = \alpha_{adopt} + \beta_{adopt} \cdot d_1$. Note that we do not include the possibility to adopt the estimates of the peers that were not the nearest, as this rarely happened in our data (Fig. 2) and would make the model overly complicated. Finally, the probability to *compromise* is given by: 1 - *P*(keep) - *P*(adopt). In our model fitting procedure, we explore the values of the *α* and *β* parameters to find the mixture of the probabilities *P* that best predict the experimental data. Our fitting procedure was constrained to exclude theoretically impossible cases of P(keep) + P(adopt) > 1. The model code is available via https://osf.io/rmcuy/.

Implementation of *compromising*. We model compromising as a Bayesian updating process [17–20] (cf. Fig. 3a). In this process, individuals weigh personal information (their own first estimate; E_1) and social information (the peer estimates; $X_{1\text{-}3}$) to produce an updated estimate (their second estimate; E_2; cf. Fig. 3a). We represent personal and social information as probability density distributions with means at the observed estimates (E_1 and $X_{1\text{-}3}$). Each of these distributions has a variance, which indicates subjective uncertainty associated with the estimate. This uncertainty is inversely related to the weight an individual will assign to that estimate in the updating process. For example, a very uncertain piece of social information (with a high variance assigned to it) will not much affect beliefs, while a very certain piece of social information (with low variance assigned to it) might cause a substantial shift in beliefs. In our model of compromising, we are interested in how the assignment of these variances that individuals assign to a piece of social information depends on (*i*) its degree of agreement with the individual's personal information (E_1), i.e., *confirmation-based weighting*; and (*ii*) its degree of agreement with other pieces of social information, i.e., a *proximity-based weighting*.

We assume that an individual's initial (prior) belief E_p about the number of animals (*N*) follows a (discretised; see below) normal distribution centred around the first estimate E_1. The uncertainty of the initial belief is captured in the variance of the distribution (σ_p^2): $p(\, E_p \mid N\,) \sim Norm(\, E_1, \sigma_p^2)$. For modelling the quantity judgments in our task, normal distributions are a natural choice to represent uncertainty around a point estimate. These distributions have two desirable properties. First, their probability density is highest at the centre. This seems reasonable as in our case, participants are incentivised to enter values they deem most likely. Second, the probability density is symmetrically decreasing as values are further away from the centre. Indeed, using normal distributions to represent

uncertainty round a point estimate is a common approach in Bayesian models of belief updating, including models considering social information [18].

These distributions are typically modelled as continuous density functions. Note that in our experiments, values of 'beliefs' were restricted to integer numbers from 1 to 150 (the range of the slider for entering estimates). To reflect this fact, and to make our analysis of compromising consistent with heuristics of keeping and adopting (by definition also restricted to integers), we discretised the normal distributions by calculating the relative probability of each integer from 1 to 150 and normalizing the sum of all probabilities to 1. Note that compromising generates a probability distribution of updated (second) estimates, which might include the individuals' own first estimate, and the estimate of a peer. As a consequence, compromising might result in instances of keeping or adopting.

Weighting personal and social information. Like individuals' own first estimate, we model social information (SI_s) as discretised normal distributions centred around peer estimates (X_s), with subjective uncertainty σ_s^2: $p(\, SI_s \mid N\,) \sim Norm(\, X_s \,, \sigma_s^2)$, where the subscript s indexes each peer estimate. Both σ_p^2 and σ_s^2 are free parameters indicating how much weight individuals assign to the personal and social information, with high values (i.e., more uncertainty) indicating less weight. When $\sigma_p^2 < \sigma_s^2$, participants assign more weight to their personal first estimate than to those of others. Individuals can adjust the weight they assign to social information (σ_s^2) based on the extent to which it confirms their own prior belief, and its proximity to the other two peer estimates.

Confirmation-based weighting depends on the absolute distance between a peer estimate and an individual's first estimate ($d_s = |E_1 - X_s|$). This distance impacts the uncertainty assigned to the estimate: $\sigma_s^2 = \alpha_s + \beta_{confirmation} \cdot d_s$, where α_s and $\beta_{confirmation}$ respectively determine the intercept and slope of the weighting function. Positive values of $\beta_{confirmation}$ imply that peer estimates agreeing more with an individual's first estimate receive more weight.

Proximity-based weighting of a peer estimate depends on its summed absolute distance (e.g., $\tau_1 = |X_1 - X_2| + |X_1 - X_3|$) to the other two peer estimates: $\sigma_s^2 = \alpha_s + \beta_{proximity} \cdot \tau_s$. Positive values of $\beta_{proximity}$ imply that peer estimates agreeing more with other peer estimates receive more weight. When we consider both confirmation- and proximity-based weighting simultaneously, they shape uncertainty σ_s^2 in an additive fashion.

Bayesian updating: calculating the posterior. As outlined above, we model compromising as a process of Bayesian updating. Here we formulate the updating process. Individuals have two sources of information to infer the number of animals (*N*): (*i*) The individual's personal belief about the number of animals (E_P) and social information provided by the peers (X_s), with s=1:*k*, and *k* being the group size. Personal beliefs are expressed as a discretised normal probability distribution centred around first estimate (E_1) and associated with uncertainty ($\sigma^2{}_p$):

$$p(\,E_p\mid N\,)\ \sim Norm(\,E_1\,,\sigma^2{}_p).$$

Similarly, we model social information (SI_s) as a discretised normal probability distributions centred around peer estimates X_s, with associated uncertainties ($\sigma^2{}_s$):

$$p(\,SI_s\mid N\,)\ \sim Norm(\,X_s\,,\sigma^2{}_s).$$

With only one peer (i.e. *k* = 1) we obtain the (posterior) probability of *N* animals by applying Bayes' rule:

$$p(\,N\mid\ E_p, SI_1\,)\ =\ \frac{p(\,E_p\mid N\,)\ *\ p(SI_1\mid\ N)\ *\ p(N)}{p(E_p, SI_1\mid N)}$$

Here, $p(\,N\mid\ E_p, SI_1\,)$ is the updated 'posterior' belief about the number of animals (*N*) given personal and social information (E_p and SI_1, respectively). We assume that before observing the images with the animals, individuals have no prior expectations regarding *N*. We therefore model *p*(*N*) as a uniform distribution across the response scale (bounded between 1 and 150).

When individuals observe three peers (as in our experimental conditions), a similar updating procedure is conducted with all peer estimates:

$$p(N\mid E_p, SI_1, SI_2, SI_3)\ =\ \frac{p(\,E_p\mid N\,)*p(\,SI_1\mid N\,)*\ p(\,SI_2\mid N\,)*p(\,SI_3\mid N\,)*p(N)}{p(E_p, SI_1, SI_2, SI_3\mid N)}$$

Note that the order of social information is not affecting the outcome of this updating process. In the probability density functions, we set the minimum values to $10^{-30}$to avoid outcome probabilities of zero.

Model analysis. We fitted the model using a hierarchical Bayesian inference technique implemented with *RStan* in *R*[21,22]. We used a hierarchical model structure, in which each model parameter had a higher-order prior (see Table S6). For all model parameters (except two, see below), priors were normal distributions with hyperparameters describing the mean and variance. The only exceptions to this were α_{keep} and α_{adopt}. Many participants (almost) never chose to keep their first estimate or to adopt the estimate of a peer (cf. Fig. 2f), necessitating a zero-inflated distribution for these two parameters. To account for individual differences in these two parameters, we used more flexible (non-normal) group-level distributions. In particular, we implemented $\alpha_{keep}$and α_{adopt}(continuous numbers) via a transformed parameter $\alpha_{keep} = logit\,(\alpha'_{keep})$ or $\alpha_{adopt} = logit\,(\alpha'_{adopt})$. This restricts parameters α'_{keep} and α'_{adopt} between zero and one, and allows us to describe these parameters with beta distributions, each having two hyperparameters controlling their shape (see Table S6). Further, note that for the fitting process, we centred the predictors d_s and $\tau_s$to their mean values. In the fitting procedure we ran 4 chains in parallel with 2,000 iterations each and discarded the first 1000 as burn-in. We reduced the memory load by thinning the chains with a factor of 5.

We investigated the predictive power of four model features: (*i*) keep heuristic; (*ii*) the adopt heuristic; and when compromising, (*iii*) confirmation-based weighting (assigning weight to social information depending on its distance of the personal first estimate), (iv) proximity-based weighting (assigning weight to social information depending its distance to other peer estimates). The importance of each of these features was evaluated by calculating the leave-one-out cross-validation (*looic*; [23]) of the models containing all possible combinations of these features (16 in total; Table S3). We quantified the importance of a feature by calculating the average reduction of the *looic* when the feature was included rather than excluded (cf. Fig. 2b of the main text). In the main text (e.g. Fig. 3c-e) we focus on the results of the best-fitting model, i.e., the model with the lowest *looic* (see Table S3). Visual inspection of Markov chains and the Gelman Rubin statistic $\hat{R}$ indicated that all Markov chains of all of the 16 investigated models converged.

Parameter recovery analysis. To ensure that all model parameters are identifiable and influence judgments in an interpretable manner, we conducted a parameter recovery analysis. Specifically, we drew eight sets of model parameters with a quasi-random number generator using the sobol sequence (from the *R* package *randtoolbox* [24]) to ensure a uniformly covered multidimensional parameter space within a reasonable parameter ranges. We used the following ranges: ${\sigma^2}_p$varies within [3,15], $\alpha_s$within [7,20], $\beta_{confirmation}$within [-0.5,0.5], $\beta_{proximity}$within [-0.5,0.5], α'_{keep}within [0,0.6], β_{keep}within [-0.5,0.1], α'_{adopt}within [0,0.6], β_{adopt}within [-0.5,0.1].

For each parameter set, we generated second estimates based on first estimates and observed social information from our four experimental conditions. We also used the same set of stimuli and the same number of individuals who participated in our experiment. This allows us to test whether the hierarchical implementation and sample size allow for an accurate identifiability of model parameters. The generated dataset thus consists of 100 participants with 20 synthetic second estimates each. We then fitted the model for each parameter set using the identical analysis as described in the main text. We report the mean and the 95% credible interval (CI)of the group-level parameter estimate. To compare input parameters and estimates of α_{keep} and α_{adopt}we used the mean of the group-level beta distribution $\mu = \frac{\alpha}{\alpha + \beta}$. We measured the relationship of input and recovered parameters by calculating Spearman's correlation coefficient. For all parameters we find a strong positive correlation between input and recovered parameters indicating that the empirically found parameters describe distinguishable aspects of judgements and are interpretable in their magnitude (Fig. S9).

Model predictions. We generated the predictions of the best-fitting model (red lines and diamonds in Fig. 1f and Fig. 2 of the main text, respectively) by calculating the probability density function for each participant in each round. This density function was based on the mean posterior parameter estimates for the participant (see Table S4 for characteristics of the parameter group-level distributions), their experimentally observed first estimate (E_1) and the displayed social information (X_S) in that round. To account for stochasticity, the model predictions in Figure 1f (red line) and Figure 2 (red diamonds) are based on 10 samples of estimates from each density function. To analyse the prediction of the best-fitting model for the experimental conditions and 'filler' rounds, we once sampled from each density function of each round and participant and calculated the actual and predicted individual-level mean adjustment and keep proportions (Fig. S6). To verify that the model parameters are distinguishable and identifiable, we conducted extensive parameter recovery analyses (Fig. S9), confirming that the model allows for reliably recovering all of its parameters.

3c. Simulations

In each of the settings shown in Fig. 4 of the main text, agents were endowed with adjustment strategies whose parameter values were sampled from the group-level distributions from the best-fitting model (Table S4), assuming no correlations between the parameters. Inspection of the joint posterior distributions of means shows that this assumption is generally justified (for details, see Table

S5; Fig. S9; sampling from the individual-level parameters from our experiment does not lead to observable differences in simulation outcomes shown in Fig. 4). For each agent, we assumed their first estimate to be 50, and simulated their adjustment given the social information in the respective social environment. For Fig. 4, the social environment consisted of ten pieces of social information, either agreeing (i.e., an estimate of 50) or disagreeing (i.e., an estimate of 65) with the focal agent. For Fig. 4d and 4e we sampled the parameters for confirmation-based weighting from the higher and lower half of the group-level distribution of $\beta_{confirmation}$, respectively. The individuals' first estimate in these settings was 55 (that is, in between the two clusters of estimates, but closer to the lower cluster).

4. **Experimental materials**

The experimental session consisted of 3 blocks (referred to as 'Task I, II and III' in the instructions for participants), the order of which was randomized across participants.

Block 1: Participants observed an image and made their first estimate of the number of animals on it. Then, they could observe the estimate of *three* participants who completed the task before, and then make a second estimate. Participants completed five rounds for each of the experimental conditions (cf. Fig. 1e of the main text), plus 10 'filler rounds' in which they observed three randomly drawn previous participants. So, in this block, participants completed 30 rounds in total; the participants' responses in the experimental rounds of this block are the main focus of this study

Block 2: Like in Block 1, participants observed an image and made their first estimate of the number of animals on it. Then, they could observe the estimate of *one* participant who completed the task before, and then make a second estimate. This was repeated for five rounds (with a new image showing another species of animal in every round). NB: the data for this control condition is not the focus of this paper, and therefore its results are not presented here.

Block 3: Participants did *not* observe an image, but had to make an estimate based on the estimates of four participants who completed the task before. The five rounds of this task mimicked the four experimental conditions plus a random round (see Experimental Design in the main text).

Below we show screenshots of the experiment. We add notes in between screenshots where appropriate, for clarification and to prevent repetitiveness.

Welcome

In this HIT we ask you to complete **three tasks** (Task I, Task II and Task III).
During these tasks you can earn points. The number of points you earn will depend on your responses.
In each task, you can earn up to **100 points.**

At the end of this HIT, your points from Task I, Task II and Task III will be added up and converted into your bonus (**100 points = $1.00**).

Your bonus will be paid on top of your guaranteed participation fee of **$4.50**.
Upon completion, you will receive a random code to collect your payment on MTurk.

In total, this HIT will take about 30-40 minutes. Please complete it without interruptions.
We finalize this HIT with a brief questionnaire.

Click below to proceed to the task instructions.
Please read these instructions carefully.

Continue

HIT description

In each of the tasks in this HIT you have to make **estimates**.
The number of points you earn depends on **how accurate** your estimates are.

Please click below to continue to Task I.

Continue

Task I: instructions 1 of 3

This task consists of **30 rounds**.

At the beginning of each round, you will observe an image showing a number of animals.

For example:

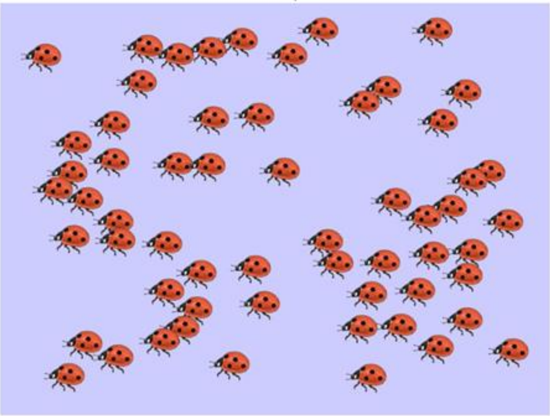

The image will disappear after 6 seconds, upon which **you have to estimate how many animals were displayed.**
The more accurate your estimate, the more points you can earn. We explain this later.

Continue

Task I: instructions 2 of 3

Once the image has disappeared, you have to enter your estimate of how many animals were displayed, by using a slider.
This is your estimate for **part A** of a round.

Once you have entered your estimate, **part B** of the round begins.
You can observe the *part A estimate of three other MTurkers.*

Over 100 MTurkers participated in a previous session in which they completed this task.
In each round, you can observe the part A estimate of **three** of these previous MTurkers.

The previous MTurkers saw the same image as you. They also saw it for **6 seconds**.
After the image disappeared, they also had to **estimate** how many animals were displayed.
They could also earn a higher bonus if their estimate was more accurate.

You then have to **enter a second estimate**.
You can enter the **same** estimate as in part A, or adjust it as you wish.
This is your estimate for **part B** of a round.

Note: please enter your estimates within the time limit on your screen.
If you do not make your estimate and press 'Continue' before the timer reaches zero,
you will be removed from the HIT and we will not be able to pay you!

Once you have entered your part B estimate, the round is over and a new round begins.

Continue

Go back

Task I: instructions 3 of 3

Your bonus earnings

The more accurate your estimates, the more points you can earn in this task.
At the end of this HIT, the points you earn are converted into your bonus earnings.
Your bonus for this task is calculated as follows.

Once you have completed this HIT, the computer will randomly select 1 of the 30 rounds.
If you estimated the number of animals ***exactly right*****, you earn 100 points.**
For each number that you are off, we subtract 5 points.
The number of points you earn cannot become negative.

For example, if the actual number of animals in the image was 60, and your estimation was 53, you were 7 off.
This would mean that we subtract 7 x 5 = 35 points. Your earnings for that estimate would be 100 - 35 = 65 points.

Click 'Continue' if you understood your task.
A brief quiz will follow to check your understanding.

Continue

Go back

Check for understanding

To check your understanding of the task, please indicate for each of these statements whether they are correct or incorrect.

In each round of this task you will view an image. You have to estimate how many animals were displayed in it.

correct | incorrect

Once you have entered your estimate, the round is over.

correct | incorrect

Once you have entered your estimate, you can observe the estimate of three other MTurkers who completed this task before. You can then make a second estimate.

correct | incorrect

The more accurate your estimates, the more points you can earn.

correct | incorrect

Continue

Back to instructions

Participants had to answer the check for understanding correctly before proceeding to the task.

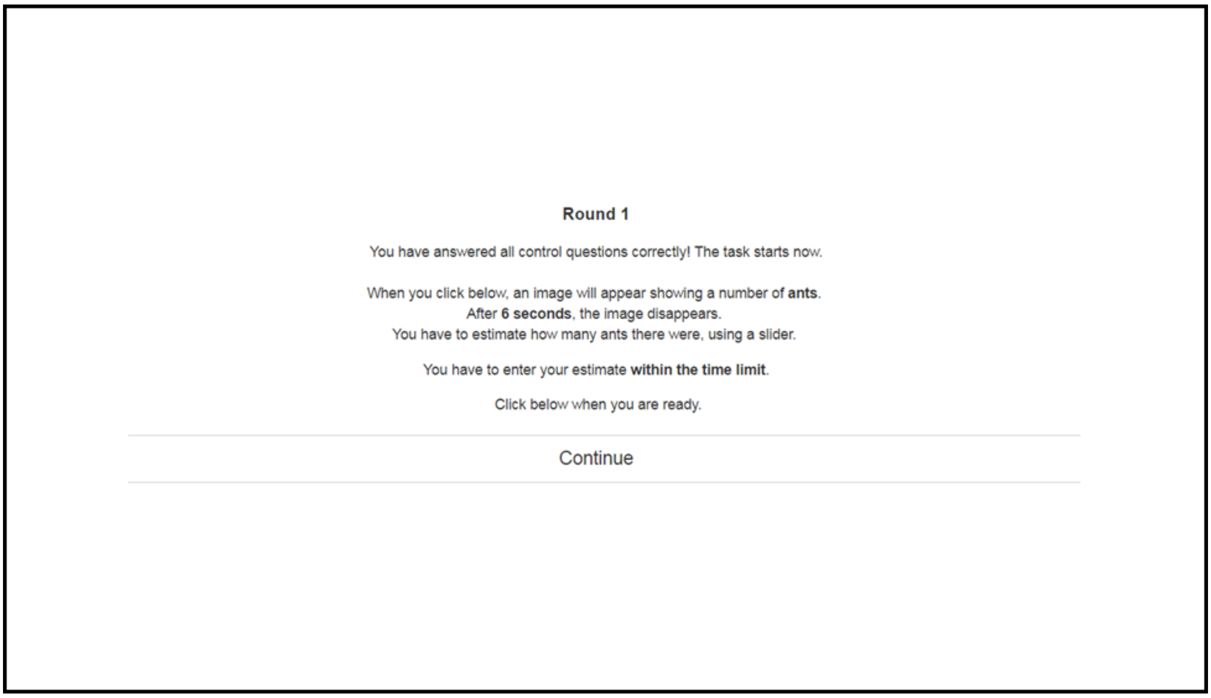

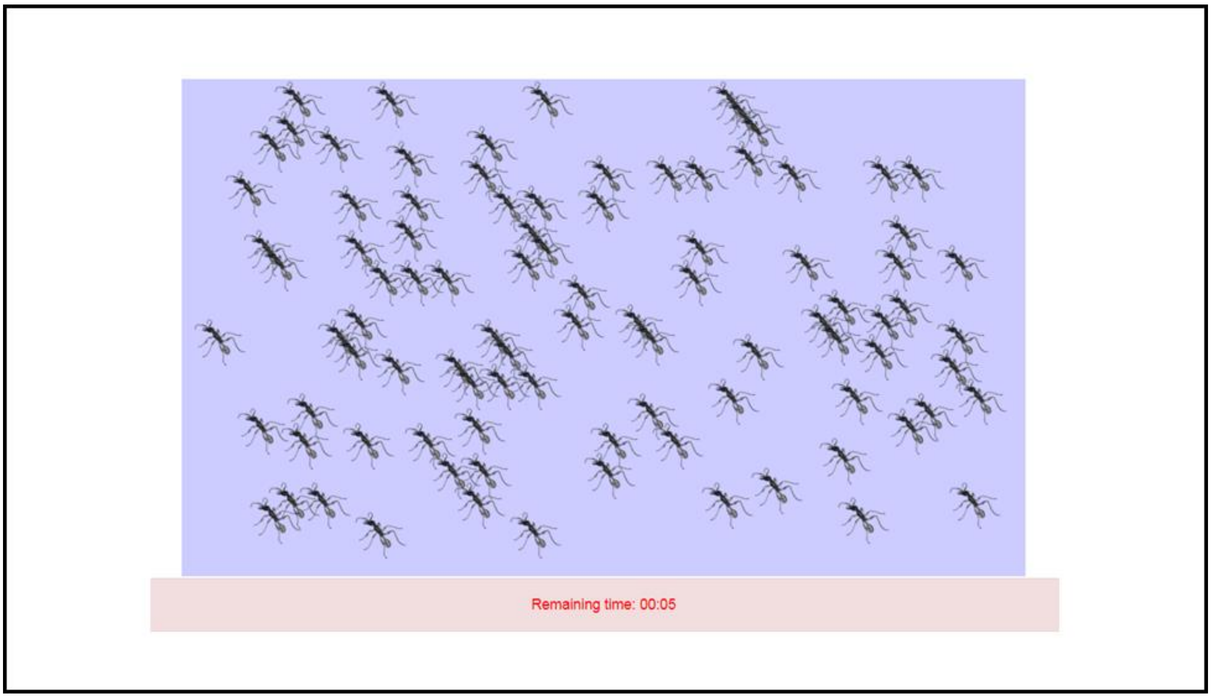

Participants could view the images for 6 seconds. We used five species of animal on the images. Across rounds, the sequence of shown species was fixed: ants-bees-flamingos-cranes-crickets-ants-bees-flamingos-cranes-crickets-ants-etc.

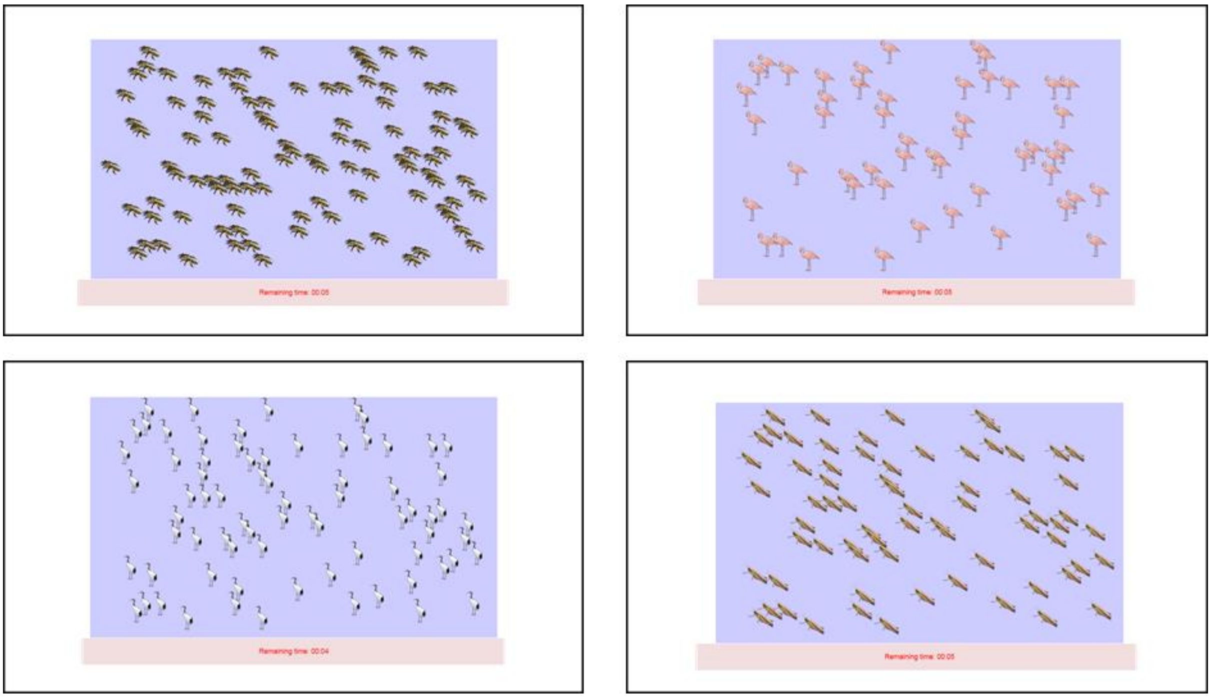

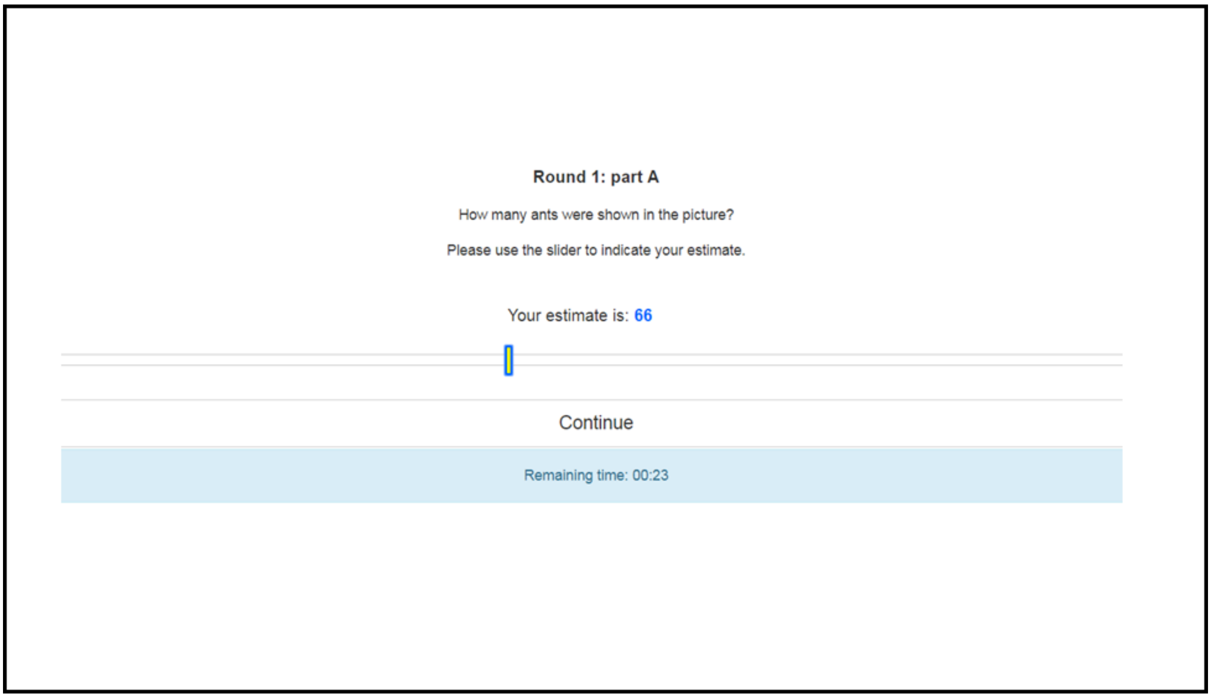

This is the decision screen for entering the *first estimate*. Participants used the slider to indicate their estimate. The slider was limited at 1 and 150 (NB: the number of animals shown in the images ranged from 50-100), and initialized at the left hand side without showing a running number. While moving the slider, the running number was shown in blue. Participants could not proceed to the next screen without moving the slider.

We now show the screens in which participants entered their second estimates. In these screens, participants observed social information in the form of the estimates of three others who completed the task before. As described in Fig. 1d of the main text and in the Methods, we manipulated social information such that the conditions differed in the variance and the skewness of the distribution of social information, while holding fixed the mean deviation from a participant's first estimate (to the extent that the pre-recorded pool of information allowed; see Methods for details and Fig. S7 for distributions of the three peers in each condition).

The following five screenshots show an example of this screen for each of the experimental conditions (plus the random condition for the 'filler' rounds). For the sake of exposition, we have filled out '60' as the first estimate in each of these rounds when making the screenshots. Note that the order of the rounds of the experimental conditions were randomized, and were intermixed with filler rounds (in which social information was randomly drawn from the pre-recorded pool of estimates). After the five screenshots, we collate the sliders from a full sequence of screens in which participants entered their first estimates. This collation aims to illustrate participants' experience in the task.

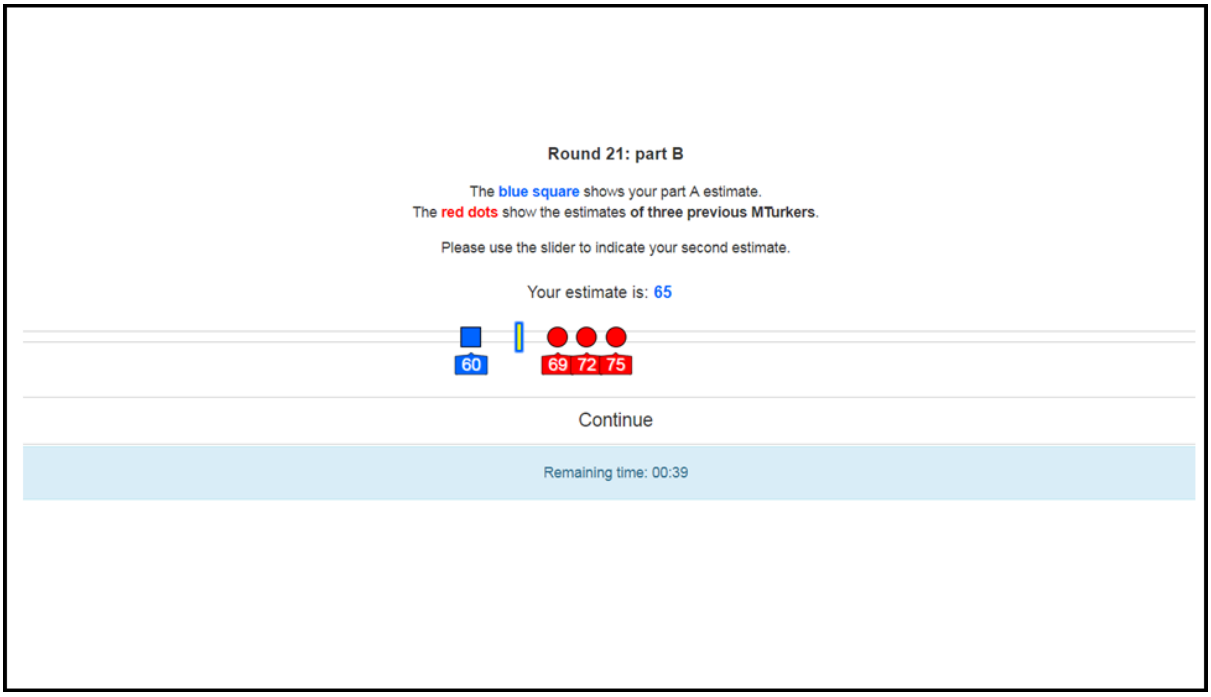

Low variance, no skewness (LN); peers strongly agreed with each other.

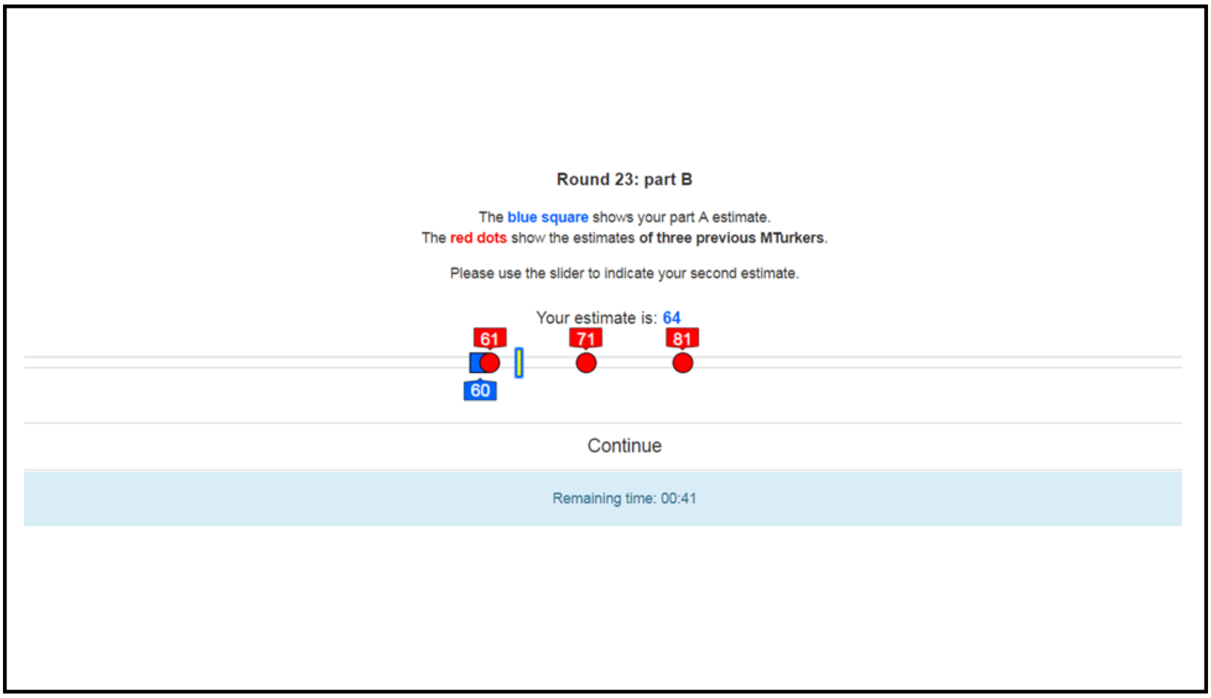

High variance, no skewness (HN). Peers disagreed amongst each other.

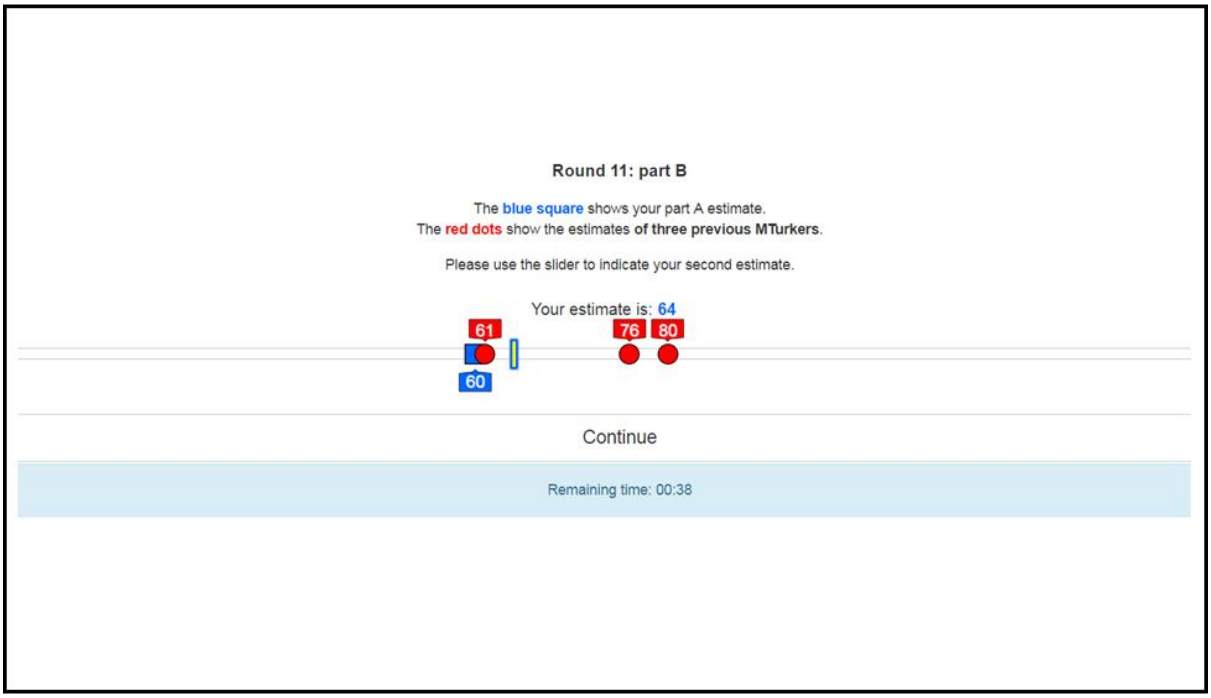

High variance, cluster far from E_1 (HF). One peer strongly agreed with the participant, while two other strongly disagreed.

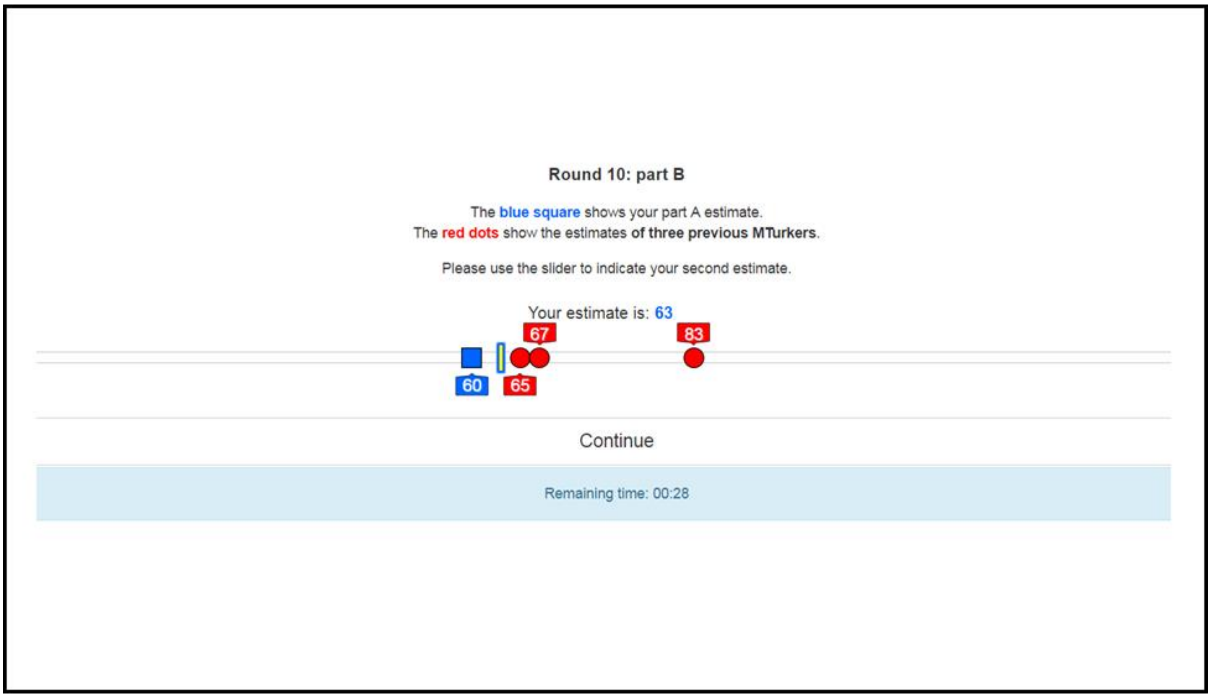

High variance, cluster close to E_1 (HC). Two peers showed moderate agreement with the participant, while one peer strongly disagreed.

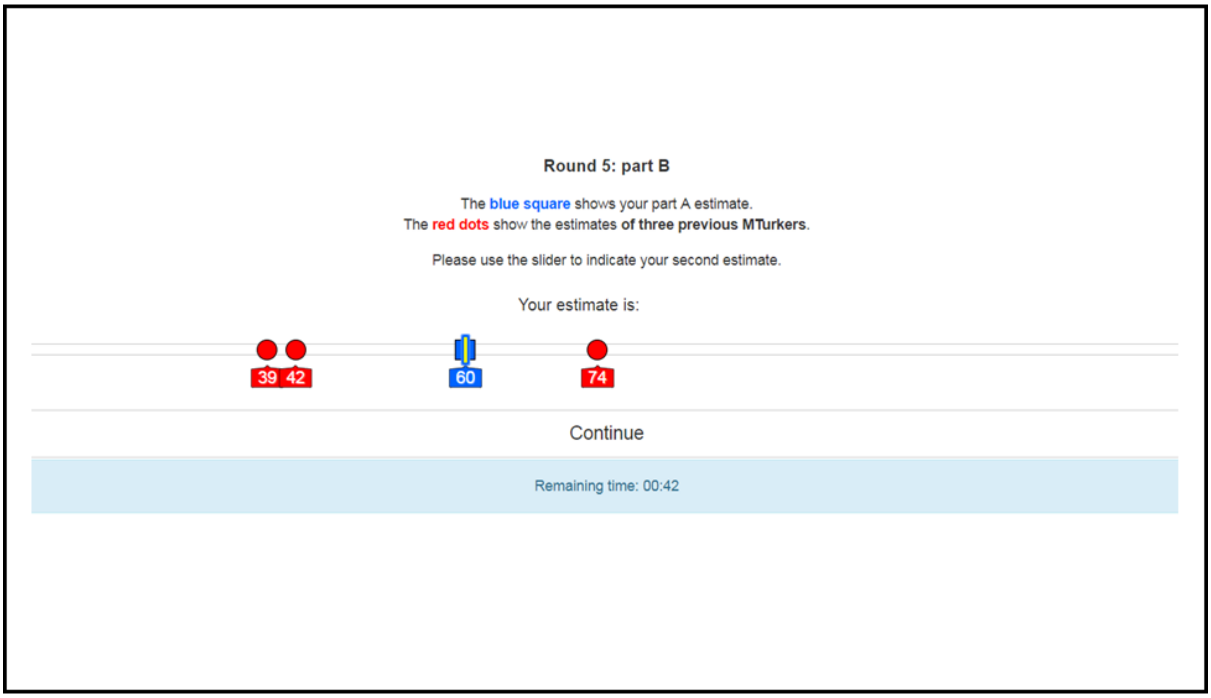

Random condition. Peer estimates were drawn randomly from the pre-recorded pool.

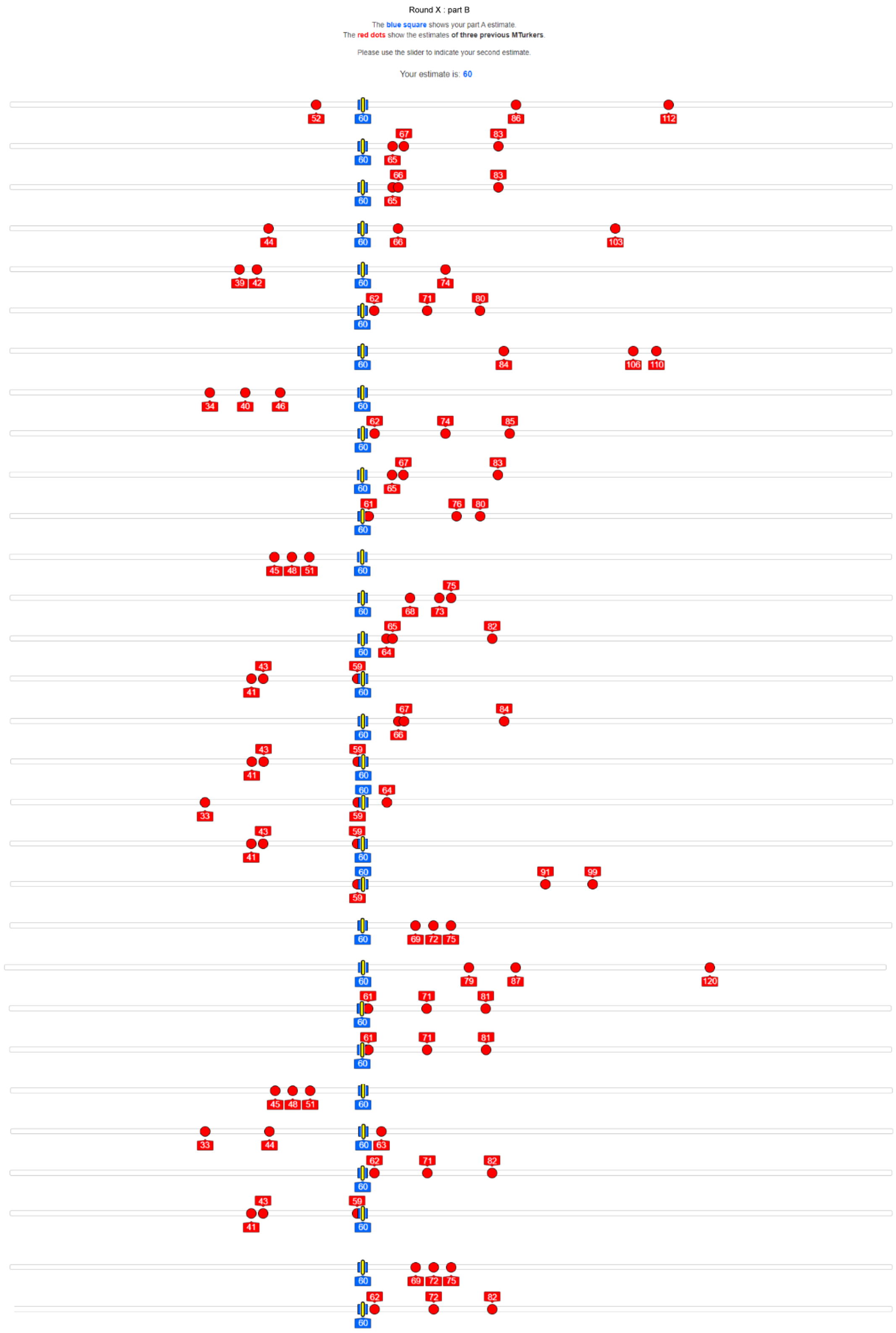

Collation of sliders from the screens of 30 rounds, in which participants entered their second estimates. Note that in the experiment, these screens were not shown right after each other: within a round, the screens showing social information were preceded by screens with a stimulus, and a screen for entering their first estimate. For illustration purposes, we here always presume the first estimate to be 60. Actual data shows more varied first estimates for each participant, and, as a consequence, social information also differed between rounds of the same treatment. It therefore

seems rather unlikely that participants identified a regular pattern over the course of the experiment (which would undermine participants' belief in the veracity of the social information, and might affect social information use). This idea is further supported by a regression fitted to adjustments in individual rounds (Table S7). In this model, 'round number' did not predict adjustments, suggesting that participants' trust in the social information displayed to them did not decrease over the course of the experiment.

After completing the 30 rounds of this block (5 rounds for each experimental condition, plus 10 filler rounds), participants proceeded to the next block.

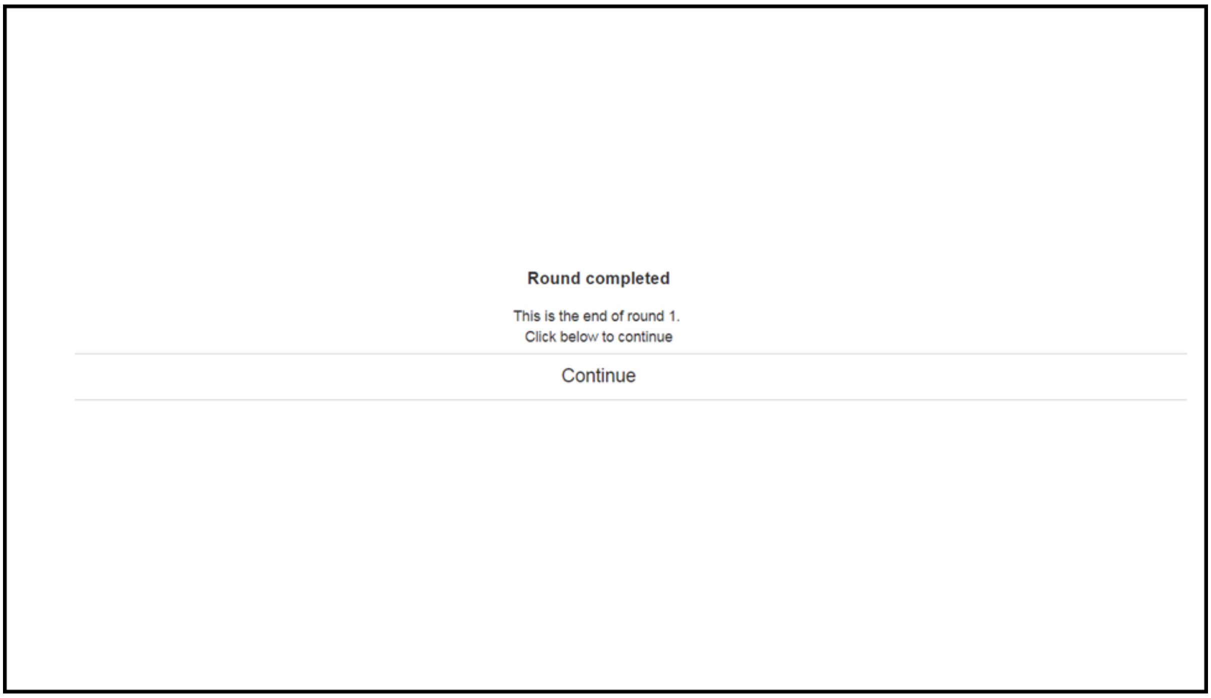

After the 30 rounds of this task were completed, this Block ended, and participants proceeded to Block 2 (referred to in the instructions as 'Task II').

End of Task I

You have completed this task. You will be able to see your earnings after you completed all three tasks and the questionnaires.
Click the button to proceed.

Go to next Task

Task II: instructions 1 of 3

This task consists of **5 rounds**.

At the beginning of each round, you will observe an **image** showing a number of animals.
For example:

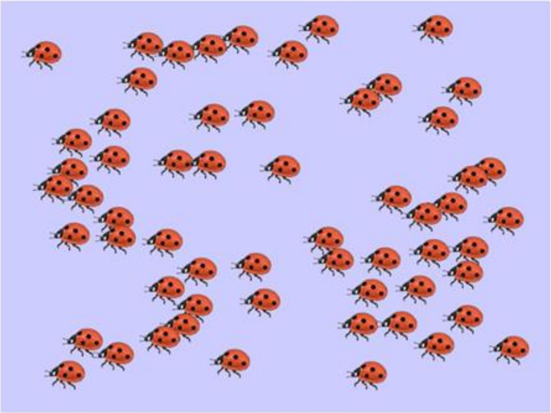

The image will disappear after 6 seconds, upon which **you have to estimate how many animals were displayed.**
The more accurate your estimate, the more points you can earn. We explain this later.

Continue

Task II: instructions 2 of 3

Once the image has disappeared, you have to enter your estimate of how many animals were displayed, by using a slider.
This is your estimate for **part A** of a round.

Once you have entered your estimate, **part B** of the round begins.
You can observe the *part A estimate of another MTurker.*

Over 100 MTurkers participated in a previous session in which they completed this task.
(These are different MTurkers from those you could observe in the previous task.)
In each round, you can observe the part A estimate of one of these previous MTurkers.

The previous MTurkers saw the same image as you. They also saw it for **6 seconds**.
After the image disappeared, they also had to **estimate** how many animals were displayed.
They could also earn a higher bonus if their estimate was more accurate.

You then have to **enter a second estimate**.
You can enter the **same** estimate as in part A, or adjust it as you wish.
This is your estimate for **part B** of a round.

*Note: please enter your estimates **within the time limit** on your screen.*
If you do not make your estimate and press 'Continue' before the timer reaches zero,
you will be removed from the HIT and we will not be able to pay you!

Once you have entered your part B estimate, the round is over and a new round begins.

Continue

Go back

Check for understanding

To check your understanding of the task, please indicate for each of these statements whether they are correct or incorrect.

In each round of this task you will view an image. You have to estimate how many animals were displayed in it.

correct | incorrect

Once you have entered your estimate, the round is over.

correct | incorrect

Once you have entered your estimate, you can observe the estimate of another MTurker who completed this task before. You can then make a second estimate.

correct | incorrect

The more accurate your estimates, the more points you can earn.

correct | incorrect

Continue

Back to instructions

Again, participants had to complete all items correctly before they could proceed to the task.

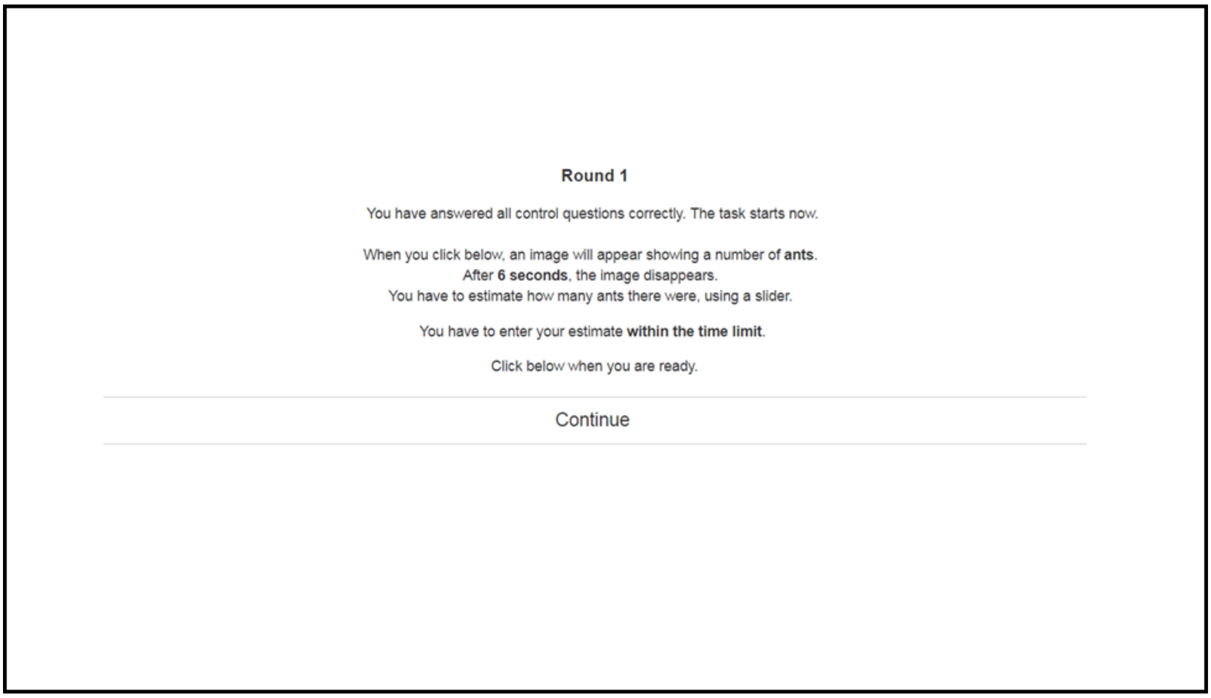

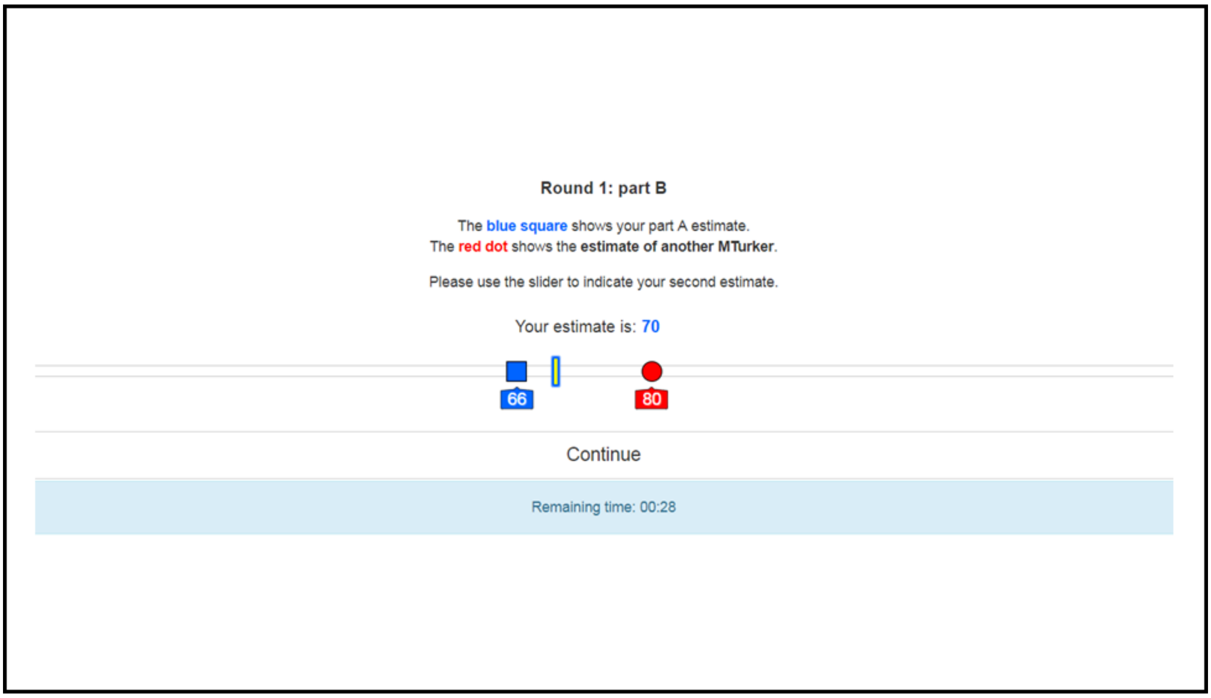

The screen for entering their first estimate was identical to the screen in the previous Block. In this one-peer (control) condition, participants could observe a single peer (here shown in red). The participant's first estimate is shown as a blue square. Participants used the slider to enter their second estimate.

End of Task II

You have completed this task. You will be able to see your earnings after you completed all three tasks and the questionnaires.
Click the button to proceed.

Go to next Task

Task III: instructions 1 of 4

This task consists of **5 rounds**.

In each round, you have to make an estimate, based on estimates of other MTurkers.
Specifically, in a HIT we conducted recently, we asked MTurkers to view 5 pictures.
Each picture displayed a number of animals.
For example:

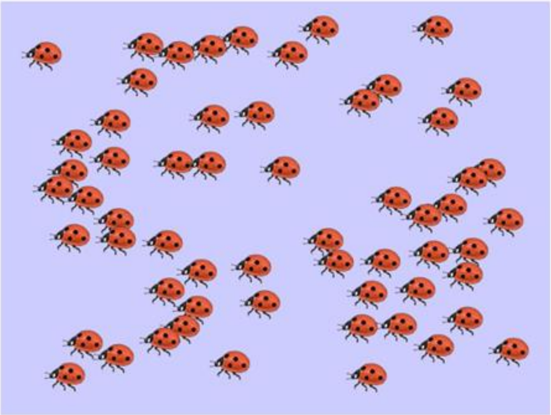

In each round, the image disappeared **after 6 seconds**, upon which the previous MTurkers had to estimate how many animals were displayed.
The more accurate their estimates, the more points they could earn.

Continue

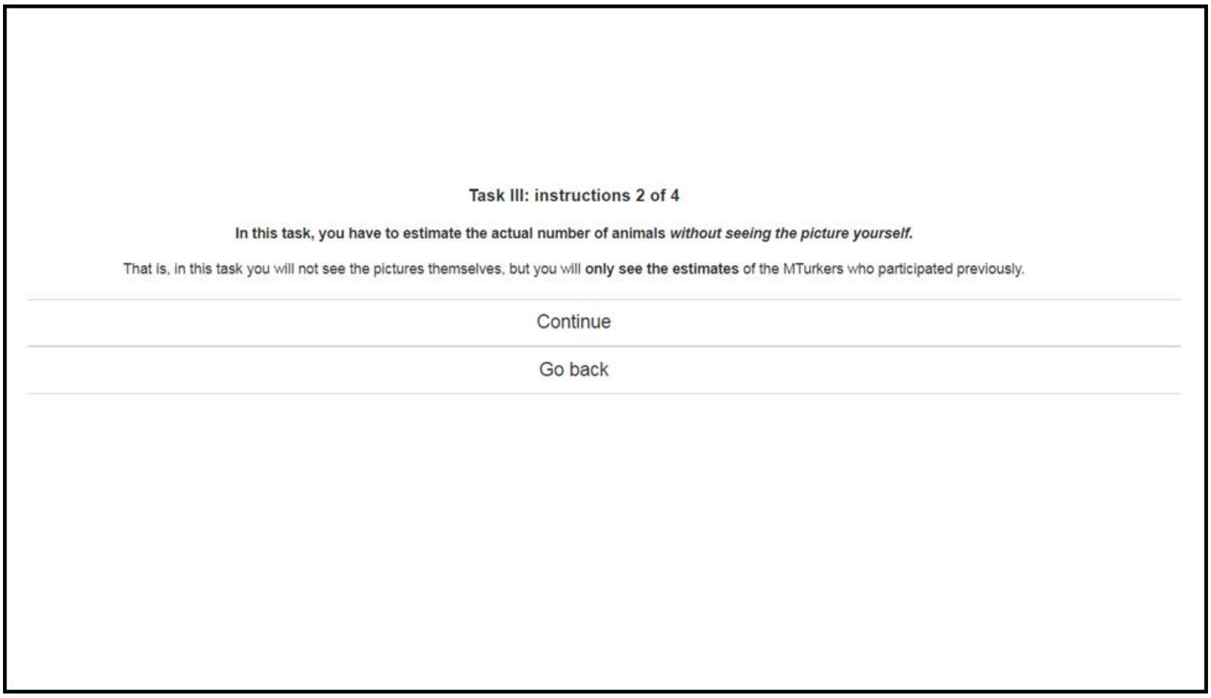

Task III: instructions 2 of 4

In this task, you have to estimate the actual number of animals *without seeing the picture yourself.*

That is, in this task you will not see the pictures themselves, but you will **only see the estimates** of the MTurkers who participated previously.

Continue

Go back

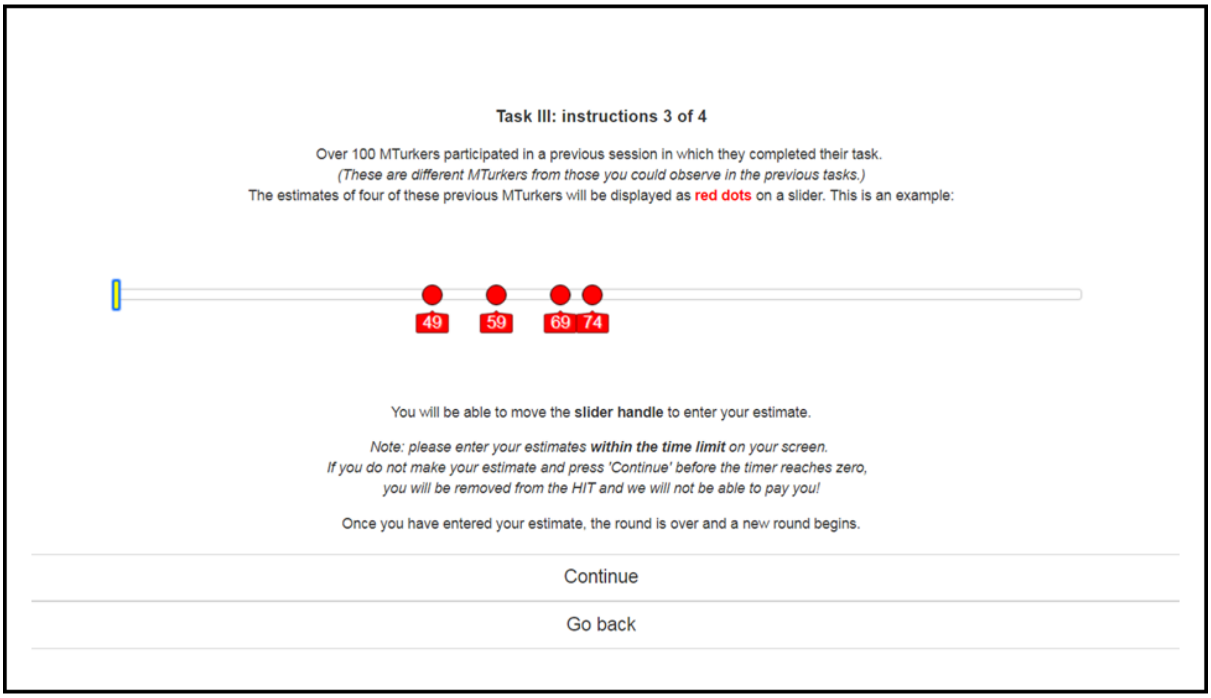

Task III: instructions 3 of 4

Over 100 MTurkers participated in a previous session in which they completed their task.
(These are different MTurkers from those you could observe in the previous tasks.)
The estimates of four of these previous MTurkers will be displayed as **red dots** on a slider. This is an example:

You will be able to move the **slider handle** to enter your estimate.

*Note: please enter your estimates **within the time limit** on your screen.*
If you do not make your estimate and press 'Continue' before the timer reaches zero,
you will be removed from the HIT and we will not be able to pay you!

Once you have entered your estimate, the round is over and a new round begins.

Continue

Go back

Task III: instructions 4 of 4

Your bonus earnings

The more accurate your estimates, the more points you can earn in this task.
At the end of this HIT, the points you earn are converted into your bonus earnings.
Your bonus for this task is calculated as follows.

Once you have completed this HIT, the computer will randomly select 1 of the 5 rounds.
If you estimated the number of animals ***exactly right***, **you earn 100 points.**
For each number that you are off, we subtract 5 points.
The number of points you earn cannot become negative.

For example, imagine that the actual number of animals in the image that the previous MTurkers saw was 60.
If your estimation was 53, you were 7 off.
This would mean that we subtract 7 *x* 5 = 35 points. Your earnings for that estimate would be 100 - 35 = 65 points.

Click 'Continue' if you understood your task.
A brief quiz will follow to check your understanding.

Continue

Go back

Check for understanding

To check your understanding of the task, please indicate for each of these statements whether they are correct or incorrect.

In each round of this task you have to estimate how many animals were shown to previous MTurkers.

correct | incorrect

In each round, you will see the estimates of one MTurker who saw the same picture.

correct | incorrect

In each round, you will see the estimates of four MTurkers who all saw the same picture.

correct | incorrect

Like yourself, the previous MTurkers could earn more points the more accurate their estimates were.

correct | incorrect

Continue

Back to instructions

Again, participants had to complete all items correctly before they could proceed to the task.

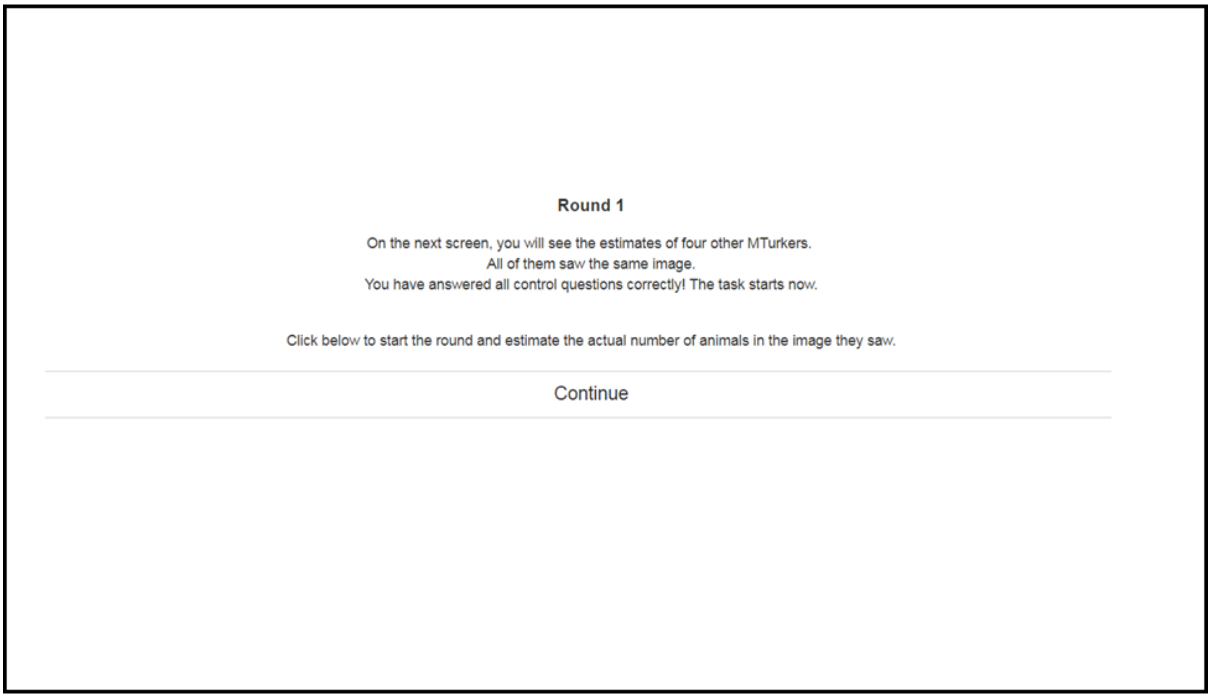

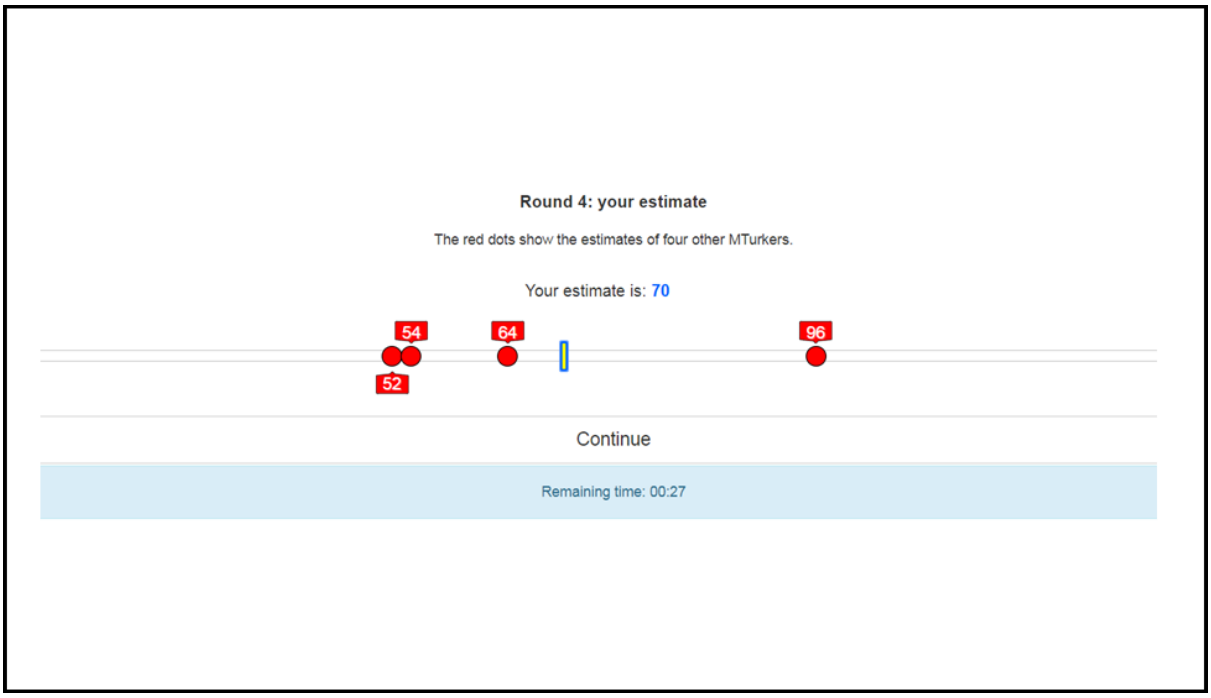

Participants did not observe an image but just observed 4 peer estimates and made their first estimate with the slider. The conditions in this block emulated the conditions in the three-peer conditions (see Methods in the main text for details). This screenshot and the next show two examples.

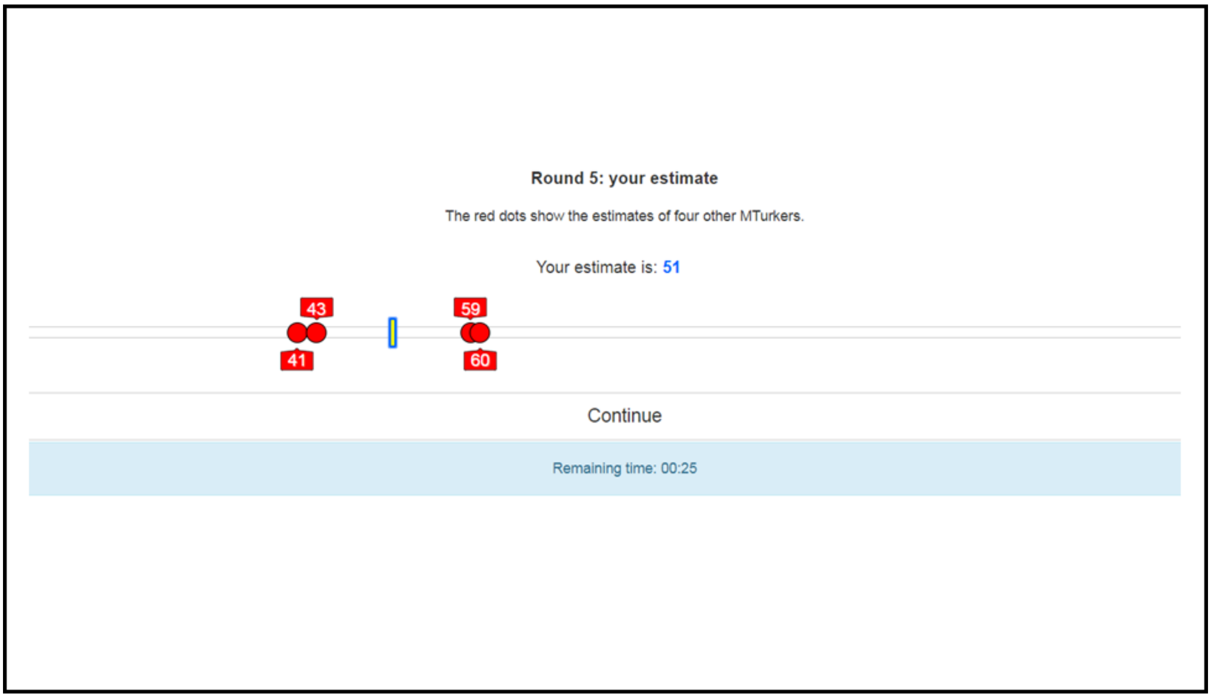

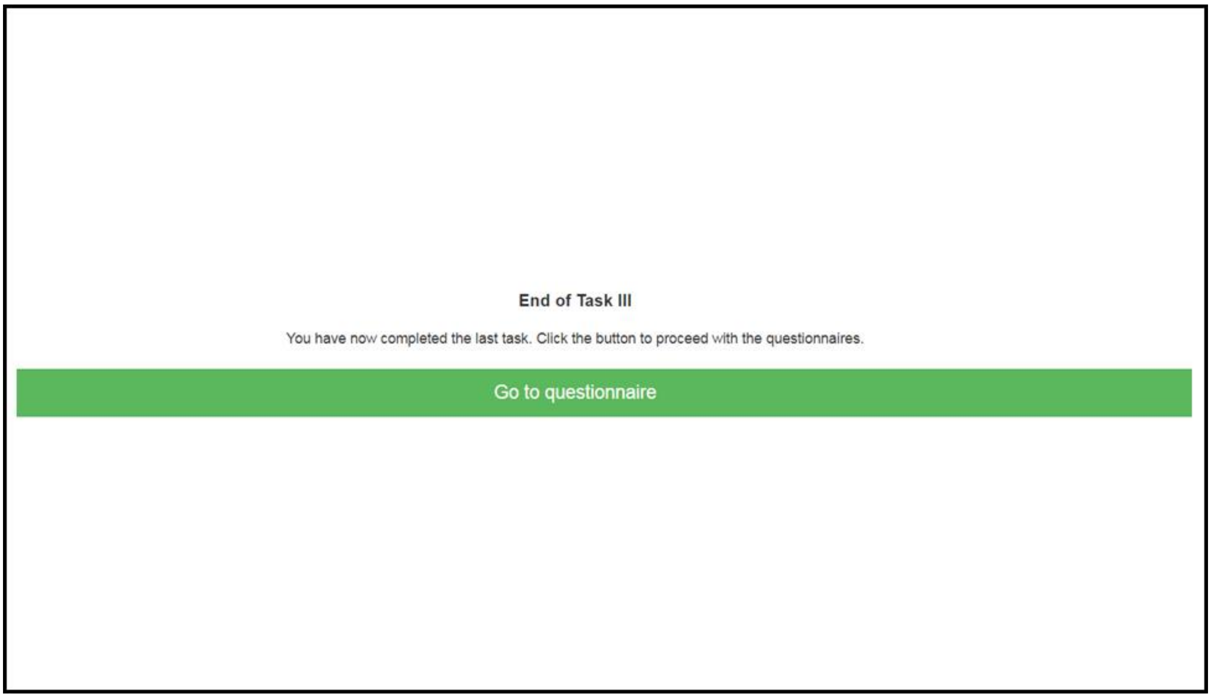

After completing the third Block, participants proceeded to a questionnaire, in which we measured age and gender (along with some other items not reported here). Finally, participants were shown their payoffs for each of the blocks.

Earnings Task I

Your earnings are calculated as follows.

The computer randomly selected round **12**.
In that round, you estimated how many **bees** there were in the image.

Your estimate in that case was **53**.
The actual number of bees in the image was **59**.

This means that your estimate was **6** off the actual number.
As a consequence, we subtract 6 x 5 = 30 Points.

You have earned 100 - 30 = **70 points**.
These Points are worth **$0.70**.

This is your bonus for this Task.

Click continue to see your bonus for the other tasks.

Continue

Earnings Task II

Your earnings are calculated as follows.

For Task I , the computer randomly selected round **3**.
In that round, you estimated how many **flamingos** there were in the image.

Your estimate in that case was **75**.
The actual number of flamingo in the image was **59**.

This means that your estimate was **16** off the actual number.
As a consequence, we subtract 16 x 5 = 80 Points.

You have earned 100 - 80 = **20 points**.
These Points are worth **$0.20**.

This is your bonus for this Task.

Click continue to know your bonus for the other tasks.

Continue

Earnings Task III

Your earnings are calculated as follows.

The computer randomly selected round **2**.
In that round, you estimated how many **animals** there were in the image that other MTurkers saw.

Your estimate in that case was **45**.
The actual number of bees in the image was **77**.

This means that your estimate was **32** off the actual number.
As a consequence, we would have subtracted 32 x 5 Points.
However, as stated in the instructions, your number of Points cannot become negative, so we subtract 100 Points.

You have earned 100 - 100 = **0 points**.
These Points are worth **$0.00**.

This is your bonus for this Task.

Click continue to know your bonus for the other tasks.

Continue

Total earnings

This is the end of this HIT. Your earnings are calculated by adding up your Points from Task I, Task II Task III:

20 (from Task I) *plus* 70 (from Task II) *plus* 0 (from Task III)= **90 Points**.

These Points are worth **$0.90**. This is your bonus for this HIT.
Note that any bonus you earn will be paid on top of your guaranteed participation fee of **$4.50**.

To collect your payment for the HIT you just completed, please fill out the below code on MTurk.

1000108

Once you have done that, you can close this window.
Thank you for your participation, and we hope you enjoyed participating in our HIT.

5. Supplementary References

1. Molleman L, Kurvers RHJM, van den Bos W. 2019 Unleashing the BEAST: a brief measure of human social information use. *Evolution and Human Behavior* (doi:10.1016/j.evolhumbehav.2019.06.005)

2. Jayles B, Kim H, Escobedo R, Cezera S, Blanchet A, Kameda T, Sire C, Theraulaz G. 2017 How social information can improve estimation accuracy in human groups. *Proceedings of the National Academy of Sciences* , 201703695.

3. Moussaïd M, Kämmer JE, Analytis PP, Neth H. 2013 Social influence and the collective dynamics of opinion formation. *PloS one* **8**, e78433.

4. Bürkner P-C. 2017 brms : An R Package for Bayesian Multilevel Models Using Stan. *Journal of Statistical Software* **80**. (doi:10.18637/jss.v080.i01)

5. Nakagawa S, Schielzeth H. 2010 Repeatability for Gaussian and non-Gaussian data: a practical guide for biologists. *Biological Reviews* **85**, 935–956. (doi:10.1111/j.1469-185X.2010.00141.x)

6. Giamattei M, Yahosseini KS, Gächter S, Molleman L. 2020 LIONESS Lab: a free web-based platform for conducting interactive experiments online. *J Econ Sci Assoc* (doi:10.1007/s40881-020-00087-0)

7. Molleman L, Kanngiesser P, Bos W van den. 2019 Social information use in adolescents: The impact of adults, peers and household composition. *PLOS ONE* **14**, e0225498. (doi:10.1371/journal.pone.0225498)

8. Sulik J, Bahrami B, Deroy O. 2020 Social influence and informational independence. *Cognitive Science Conference Proceedings* **19**.

9. Triandis HC, Gelfand MJ. 1998 Converging measurement of horizontal and vertical individualism and collectivism. *Journal of personality and social psychology* **74**, 118.

10. Mehrabian A, Stefl CA. 1995 Basic temperament components of loneliness, shyness, and conformity. *Social Behavior and Personality: an international journal* **23**, 253–263.

11. Steinberg L, Monahan KC. 2007 Age differences in resistance to peer influence. *Developmental Psychology* **43**, 1531–1543. (doi:10.1037/0012-1649.43.6.1531)

12. Yaniv I. 2004 Receiving other people's advice: Influence and benefit. *Organizational Behavior and Human Decision Processes* **93**, 1–13. (doi:10.1016/j.obhdp.2003.08.002)

13. Schultze T, Rakotoarisoa A-F, Schulz-Hard S. 2015 Effects of distance between initial estimates and advice on advice utilization. *Judgment and Decision making* **10**, 144–172.

14. Yaniv I, Milyavsky M. 2007 Using advice from multiple sources to revise and improve judgments. *Organizational Behavior and Human Decision Processes* **103**, 104–120.

15. Yaniv I, Kleinberger E. 2000 Advice Taking in Decision Making: Egocentric Discounting and Reputation Formation. *Organizational Behavior and Human Decision Processes* **83**, 260–281. (doi:10.1006/obhd.2000.2909)

16. Scheibehenne B, Rieskamp J, Wagenmakers E-J. 2013 Testing adaptive toolbox models: A Bayesian hierarchical approach. *Psychological review* **120**, 39.

17. Adjodah D, Leng Y, Chong SK, Krafft P, Pentland A. 2017 Social Bayesian Learning in the Wisdom of the Crowd. *arXiv:1712.09960 [physics]*

18. Bang D, Frith C. 2017 Making better decisions in groups. *Royal Society Open Science* **4**, 170193. (doi:10.1098/rsos.170193)

19. Park SA, Goïame S, O'Connor DA, Dreher J-C. 2017 Integration of individual and social information for decision-making in groups of different sizes. *PLOS Biology* **15**, e2001958. (doi:10.1371/journal.pbio.2001958)

20. Toelch U, Dolan RJ. 2015 Informational and normative influences in conformity from a neurocomputational perspective. *Trends in cognitive sciences* **19**, 579–589.

21. R Core Team. 2015 *R: A Language and Environment for Statistical Computing*. Vienna, Austria: R Foundation for Statistical Computing. See http://www.R-project.org/.

22. Stan Development Team. 2018 *RStan: the R Interface to Stan. R package version 2.17. 3*.

23. Vehtari A, Gelman A, Gabry J. 2016 loo: Efficient leave-one-out cross-validation and WAIC for Bayesian models. *R package version* **1**.

24. Dutang C, Savicky P. 2013 randtoolbox: Generating and testing random numbers. *R package* , 67.
